# Supplementary material for: The Inventory of Nonordinary Experiences (INOE): Evidence of validity in the United States and India
Source: PLoS One. 2023 Jul 26;18(7):e0287780. doi: 10.1371/journal.pone.0287780 (PMC10370766; doi:10.1371/journal.pone.0287780)
Supplement: S1 Appendix — (PDF) [file pone.0287780.s001.pdf]

S1 Appendix
INOE Items, Intended Interpretations, and Version History

The aim of the Inventory of Non-Ordinary Experiences (INOE) is to gather information on experiences that might stand out for people in different cultures relative to what they consider ordinary or everyday. Because it holds open the possibility that different experiences might stand out for people in different cultures, it is not intended to measure an overall theoretical construct (e.g., religious, spiritual, paranormal, anomalous, or psychopathological experiences). Rather, the INOE identifies experiences based on phenomenological features that can be recognized across cultures and academic disciplines, and then asks follow-up questions to capture the context, the effects, and how people appraised it. The INOE separates experiences from appraisals because (1) the same appraisal may be applied to phenomenologically distinct experiences, and (2) different appraisals may be applied to phenomenologically similar experiences.

The items were developed through a review of measures that target experiences that researchers characterize as non-ordinary, regardless of the construct they purport to measure. We added experiences valorized or cultivated by various religious or spiritual traditions.

The INOE includes two sections: (1) experience items and (2) follow-up items. In the online version, respondents give yes/no responses to all the experience items. When they are finished, the items to which they responded “yes” reappear with follow up questions. Indian participants were given a choice between completing the INOE in Roman or Devanagari script.

Measures Reviewed

Sources that inspired items are indicated with abbreviations where relevant.

AEI	The Anomalous Experience Inventory [1].
AANEX	The Appraisals of Anomalous Experiences Interview Probes [2].
ASGS	The Australian Sheep-Goat Scale [3,4].
CEQ	The Creative Experiences Questionnaire [5].
DSES	The Daily Spiritual Experience Scale [6].
DES	The Dissociative Experiences Scale [7].
EDI	The Ego Dissolution Inventory [8].
KS	The Kundalini Scale [9].
MEQ	The Mystical Experience Questionnaire [10,11].
MS	The Mysticism Scale [12].
NDES	The Near-Death Experience Scale [13].
NAOS	The New Age Orientation Scale [14].
PAGE-R	The Questionnaire for Assessing the Phenomenology of Exceptional Experiences [15].
RPBS	The Revised Paranormal Belief Scale [16].
SAE	The Survey of Anomalous Experiences [17,18].
TAS	The Tellegen Absorption Scale [19,20].
TS	The Transliminality Scale [21].
O-LIFE	The “Unusual Experience” Section of the Oxford-Liverpool Inventory of Feelings and Experiences [22].

Inventory of Non-Ordinary Experiences (INOE)

Taves, A., Ihm, E., Wolf, M., Barlev, M., Kinsella, M., & Vyas, M.

Note: Nicknames such as "Joy" are for the convenience of researchers and are not part of the item.

Experience Items

EMOTION ITEMS

Because most of the Emotion items ask about phenomenological features that may be relatively commonplace, we qualify all but the last two items in this set ask if the respondent has had an experience of [an emotion] that stood out from all other such experiences. Here we discuss the intended interpretation for the "stood out" qualifier. We give the intended interpretation for the specific features individually below.

- **Intended Interpretation of "stood out":** The "stood out**"** qualifier by asking if the respondent has had a particular experience of the specified emotion (the feature) that is markedly different from all their other experiences of that specified emotion, and/or markedly different from what they consider ordinary or everyday experiences of that sort. To assess whether they understood this, we asked them to paraphrase both the feature alone (the substance paraphrase) and the feature with the qualifier (the overall paraphrase) and give an example.

- **Coding notes**: To answer the question the respondent must compare a particular instance of an emotion with their other experiences of that emotion. They may offer various indications of having done so. They may explicitly say that the experience is unique or different from any other such experiences. They may indicate this using a superlative adjective, which shows how one thing is different from others of its kind, e.g., most, deepest, biggest, best, worst. For example, they may indicate that the experience differed because it was more intense, extreme, or complete than other experiences involving the feature of interest (e.g., fear, pleasure). Alternatively, if their example attached the emotion to a relatively rare, intense, or surprising event, such as a birth, death, or accident, we inferred that they understood "stood out" as well.

1. **Joy.**
    a. I have had an experience of joy, ecstasy, or bliss that stood out from all other such experiences.
    b. Mujhe harsh, anand ya khushi ka anubhav hua hai jo baki aise anubhavo se alag ho.
    c. मुझे हर्ष, आनंद या ख़ुशी का अनुभव हुआ है जो बाकि ऐसे अनुभवों से अलग रहा हो।
    d. <u>Intended Interpretation</u>: (Oxford Languages online). Joy = a feeling of great pleasure and happiness. Ecstasy (1) = an overwhelming feeling of great happiness or joyful excitement (synonyms = bliss). Ecstasy (2) = an emotional or religious frenzy or trance-like state, originally one involving an experience of mystic self-transcendence. Bliss (1) = perfect happiness; great joy. Bliss (2) = a state of spiritual blessedness, typically that reached after death.
    e. <u>Coding Notes</u>: The first definitions of joy, ecstasy, and bliss have a great deal of overlap; they are basically synonyms. But their second definitions carry religious or spiritual connotations that take them in somewhat different directions. Any of these interpretations are fine.
    f. <u>Sources</u>: The item was inspired by items related to "joy" in the DSES, MS, NDES, MEQ30 and expanded to include "ecstasy" and "bliss" that overlap and are cultivated in some traditions, e.g. Hinduism.
    g. <u>Iteration Count & Extent of Change</u>: 1 in English, 1 in Hindi. No change.

2. **Peace.**
    a. I have had an experience of peace or wholeness that *stood out from all other such experiences*.
    b. Mujhe shanti ya purnata ka aisa anubhav hua hai jo baki aise anubhavo se kafi alag tha.
    c. मुझे शांति या पूर्णता का ऐसा अनुभव हुआ है जो बाकि ऐसे अनुभवों से काफी अलग था।
    d. Intended Interpretation: Peace is generally understood as “freedom from anxiety, disturbance (emotional, mental, or spiritual), or inner conflict; calm, tranquility” (OED, 3a); in the context of this item, it is meant to be interpreted as a harmonious feeling of ‘inner peace’ (rather than simply the absence of interpersonal hostility or violence). Wholeness is understood as the state of forming a complete and harmonious whole; unity” (Oxford Languages online).
    e. Coding Notes: Since the item used "or" any combination of these meanings is fine. Peace appears in spirituality and mysticism scales; it is valorized along with ‘wholeness’ in many religious traditions.
    f. Sources: This item was inspired by items that referred to peace, harmony, and tranquility in the AANEX, DSES, MS, NDES, MEQ30.
    g. Iteration Count & Extent of Change: 3 in English, 3 in Hindi. Minor change.
    h. First Iteration: I have had an experience of inner peace, harmony, or wholeness that stood out from all other such experiences. / Mujhe antarik shanti, ekrasata, ya purnata ka aisa anubhav hua hai jo baki aise anubhavo se kafi alag tha.
3. **Love.**
    a. I have had an experience of love that stood out from all other such experiences.
    b. Mujhe prem ka ek anokha anubhav hua hai jo baki aise anubhavo se alag tha.
    c. मुझे प्रेम का एक अनोखा अनुभव हुआ है जो बाकि ऐसे अनुभवों से अलग था।
    d. Intended Interpretation: Love is a deep feeling of affection and attachment, which includes but is not limited to romantic feelings (adapted from Oxford Language online).
    e. Coding Notes: Religious traditions often cultivate this feeling, so the feeling can be of any duration. It does, however, need to "stand out" from other such experiences. This feeling is likely most common in relation to people, but also may be felt in relation to animals or in relation to various contexts or without any apparent precipitating cause.
    f. Sources: This item was inspired by items that referred to love and/or God’s love in the AANEX and DSES.
    g. Iteration Count & Extend of Change: 5 in English, 3 in Hindi. Major change.
    h. First Iteration: I have had an experience of love or compassion that stood out from all other such experiences. / Mujhe prem aur karuna ki aisi kshan ka anubhav hua hai jo baki aise anubhavo se alag raha ho.
4. **Loss**.
    a. I have had an experience of loss (of any kind) that stood out from all other such experiences.
    b. Maine kisi chiz (vyakti ya anya vastu) ke khone ka anubhav mahsus kiya hai jo baki aise anubhavo se kafi alag tha.
    c. मैंने किसी चीज़ (व्यक्ति या अन्य वस्तु) के खोने का अनुभव महसूस किया है जो बाकि ऐसे अनुभवों से काफी अलग था।
    d. Intended Interpretation: The fact or process of losing something or someone important (adapted from Oxford Language online).
    e. Coding Notes: Examples include the death of a loved one, losing an object that has emotional or monetary value, or significant loss of physical or mental functioning.
    f. Source: This item is included as a negative emotion that may be moderated by appraisals.
    g. Iteration Count & Extend of Change: 2 in English, 2 in Hindi. Minor change.
    h. First Iteration: I have had an experience of loss that stood out from all other such experiences. / Mujhe kuch kho baithne ka aisa mehsus kiya he jo baki aise anubhavo se alag raha ho.

5. **Awe.**
    a. I have had an experience of awe, wonder, or amazement that stood out from all other such experiences.
    b. Mujhe aashcharya aur vismai bhara anubhav hua hai jo baaki aise anubhavo se kaafi alag tha.
    c. मुझे आश्चर्य और विस्मय भरा अनुभव हुआ है जो बाकी ऐसे अनुभवों से काफी अलग था।
    d. Intended Interpretation: An experience of something vast, which challenges one's ability to understand it, or goes beyond what could have been imagined previously.
    e. Coding Notes: Vastness may be physical (e.g., size) or more abstract (e.g., the extent of the Dalai Lama's compassion, the explanatory scope of the theory of evolution; see Keltner & Haidt, 2003). There is often a conscious recognition that the person needs to update their mental model or concept of the world or some aspect of it. Often involves feelings of being overwhelmed or speechless. Typical examples include expansive views of nature, witnessing great accomplishments, or hearing "big" new ideas. But any experience could count, as long as it is perceived as vast and beyond one's previous understanding of the world.
    f. Source: This item was inspired by secular spiritualities that cultivate awe and wonder.
    g. Iteration Count & Extend of Change: 1 in English, 1 in Hindi. No change.
6. **Fear.**
    a. I have had an experience of fear or horror that stood out from all other such experiences.
    b. Mujhe dar ya bhay ka aisa anubhava hua hai jo baki aise anubhavo se kafi alag tha.
    c. मुझे डर या भय का ऐसा अनुभव हुआ है जो बाकि ऐसे अनुभवों से काफी अलग था।
    d. Intended Interpretation: "An unpleasant emotion caused by the belief that someone or something is dangerous, likely to cause pain, or a threat" (Oxford Language online).
    e. Sources: This item was inspired by items that referred to fears of various sorts in the AEI and KS.
    f. Iteration Count & Extend of Change: 1 in English, 1 in Hindi. No change.
7. **Hopelessness.**
    a. I have had a feeling of hopelessness that stood out from all other such feelings.
    b. Maine aisi nirasha mehsus ki hai jo sabse alag anubhav tha.
    c. मैंने ऐसी निराशा मेहसूस कि है जो सबसे अलग अनुभव था।
    d. Intended Interpretation: A feeling of despair; no expectation of success or positive outcome (adapted from Oxford Language online).
    e. Sources: This item, which originally referred to despair rather than hopelessness, was inspired by items related to feelings of impending doom or sudden dread in AANEX and O-LIFE.
    f. Iteration Count & Extend of Change: 2 in English, 2 in Hindi. Minor change.
    g. First Iteration: I have had an experience of despair that stood out from all other such experiences. / Mujhe nirasha ka aisi anubhav hua he jo baki aise anubhavo se alag raha tha.
8. **Misfortune**:
    a. I have had an experience of misfortune that stood out from all other such experiences.
    b. Muje aise durbhagya ka anubhava hua hai jaise kisi ne mujpe panauti ya buri najar daali ho.
    c. मुझे ऐसे दुर्भाग्य का अनुभव हुआ है जैसे किसी ने मुझपे पनौती या बुरी नज़र डाली है।
    d. Intended Interpretation: an event or conjunction of events that causes an unfortunate or distressing result (Merriam-Webster online)
    e. Coding Notes: Any sort of misfortune is included as long as it stands out. Examples include the death of child, murder, rape, earthquakes, fires, etc.
    f. Source: This item was inspired by witchcraft beliefs and included as a negative emotion that may be moderated or enhanced by appraisals.
    g. Iteration Count & Extend of Change: 1 in English, 1 in Hindi. No change.

9. **Compassion**.
    a. I can recall a specific experience in which I felt compassion for the suffering of others (human or nonhuman) that stood out from all other such experiences.
    b. Muje anya jivo (manav ya aur koi bhi) ke dukh ke liye karuna ka ek vishishta anubhav hua hai jo baki aise anubhavo se alag tha.
    c. मुझे अन्य जीवों (मानव या और कोई भी) के दुख के लिए करुणा का एक विशिष्ट अनुभव हुआ है जो बाकी ऐसे अनुभवों से अलग था।
    d. Intended Interpretation: Compassion is a "sympathetic pity and concern for the sufferings or misfortunes of others" (Oxford Language online).
    e. Source: This item was inspired by traditions, such as Buddhism, that cultivate compassion.
    f. Iteration Count & Extend of Change: 5 in English, 4 in Hindi. Major change.
    g. First Iteration: I have had an experience of compassion that stood out from all other such experiences. / Mujhe karuna ke aise pal ka anubhav hua hai jo baki aise anubhavo se alag raha ho.
10. **Pleasure.**
    a. I have had an experience of pleasure that stood out from all other such experiences.
    b. Muje ek baar maze ya bhog-vilas ka vishishta anubhav hua hai jo baki aise anubhavo se alag raha ho.
    c. मुझे एक बार मज़े या भोग-विलास का विशिष्ट अनुभव हुआ है जो बाकि ऐसे अनुभवों से अलग रहा हो।
    d. Intended Interpretation: "a feeling of happy satisfaction and enjoyment" (Oxford Language online) that stands out from all other such feelings.
    e. Differential Diagnosis: **Pleasure** is defined in terms of happiness and enjoyment and is associated with things that are fun. The **Joy** item is a more extreme version of **Pleasure** that has religious or spiritual connotations in some contexts.
    f. Source: This item was inspired by the Hindu concept of *lila,* which has no direct equivalent in English but is sometimes translated as "play."
    g. Iteration Count & Extend of Change: 1 in English, 3 in Hindi. Minor change.
    h. First Iteration, if different: Muje maze ya bhog-vilas ka anubhav hua hai jo baki aise anubhavo se alag raha ho.
11. **Places (special)**.
    a. I have felt a sense of deep attachment or connection to a specific place that stood out from all other such places.
    b. Muje kisi ek vishishta sthal ya jagah ke liye atyant lagaav ya gehra rishta raha hai jo baki sthalo se alag ho
    c. मुझे किसी विशिष्ट स्थल या जगह के लिए अत्यंत लगाव या गहरा रिश्ता रहा है जो बाकि स्थलों से अलग हो।
    d. Intended Interpretation: A feeling of deep attachment or connection to a particular place.
    e. Coding Notes: It could be a local place that their community or people view as special, as is the case with many indigenous people, or a distant place that a tradition views as special, e.g., Jerusalem, Mecca, or Bodh Gaya. It could be a place they have visited, a place they once lived, or a place they imagine going in the future here on earth or elsewhere, e.g., Heaven or a distant planet. It can also be a type of place.
    f. Sources: This item was inspired by the emphasis on sacred sites in indigenous traditions and on holy places in many religions.
    g. Iteration Count & Extend of Change: 2 in English, 2 in Hindi. Minor change.
    h. First Iteration: I have felt a sense of deep attachment or connection to a specific place. / Muje kisi vishishta sthal ya jagah ke liye atyant lagaav raha hai jo baki sthalo se alag ho.

12. **Devotion (objects).**
   a. I have felt devotion or intense attachment toward an irreplaceable object or image.
   b. Kisi amoolya padaarth ya pratima (jo ajod hai aur jiski koi nakal nahi) ke liye muje atyant aadar ya lagaav hai.
   c. किसी अमूल्य पदार्थ या प्रतिमा (जो अजोड़ है और जिसकी कोई नक़ल नहीं) के लिए मुझे अत्यंत आदर या लगाव है।
   d. Intended Interpretation: A feeling of deep attachment or connection to a special object or image, or set (or type) of objects or images.
   e. Coding Notes: The word 'devotion' has religious overtones, but we are leaving whether they consider their devotion religious or not to the appraisals. Common examples might include heirlooms, pictures of loved ones, gifts from someone special, or wedding rings. People may feel objects are irreplaceable, e.g., if given to them by someone special, even if copies are available. Virtual objects, such as Avatars or Video game characters, are OK. Religious objects could include the Catholic Eucharistic wafer (or Blessed Sacrament), Eastern Orthodox icons, *murtis* (statues of Hindu deities), or New Age crystals.
   f. Source: This item was inspired by the sacralization of objects in many traditions and the common practice of setting objects apart as special.
   g. Iteration Count & Extend of Change: 2 in English, 4 in Hindi. Minor change.
   h. First Iteration: I have felt intense devotion toward an object or image. / Kisi pratima ya vastu ke liye muje atyant bhakti hai. Kya upar diya gaya anubhav aapke sath kabhi hua hai?

13. **Devotion (people).**
   a. I have felt intense devotion toward a leader or influential person.
   b. Kisi neta, guru ya prasiddh vyakti ke liye muje atyant aadar ya lagaav hai.
   c. किसी नेता, गुरु या प्रसिद्ध व्यक्ति के लिए मुझे अत्यंत आदर या लगाव है।
   d. Intended Interpretation: A feeling of deep attachment or connection to a human being that is a leader or other influential person.
   e. Coding Notes: "Devotion" can be interpreted either as a feeling e.g., of love, loyalty, respect, strong inspiration, or desire to emulate, or as an action, e.g., in some way acting out that feeling of devotion, e.g., joining the person's group. In general, we are looking for people who are not family members or part of their friendship circle, but there may be some exceptions. Generally speaking, we are looking for attachment to what the sociologists would call "charismatic" figures. The word has religious overtones, but we are leaving whether they consider their devotion religious or not to the appraisals. Common examples might include political leaders (Bernie Sanders [in 2016], Trump, Modi), religious figures, such as teachers, saints, or gurus; and popular culture figures, such as movie stars, singers, celebrities, and social media stars.
   f. Source: This item was inspired by the sacralization of people (e.g., as shamans, saints, or gurus) in many traditions and the common practice of setting charismatic leaders apart as special.
   g. Iteration Count & Extend of Change: 3 in English, 3 in Hindi. Minor change.
   h. First Iteration, if different: I have felt intense devotion toward a leader, teacher, or prominent person.

SENSORY / BODY ITEMS

14. **Light(s).**
   a. I have seen light or lights that appeared to have no ordinary physical source.
   b. Maine aisi roshni dekhi hai jiski koi sadharan vajah nahi thi.
   c. मैंने ऐसी रोशनी देखी है जिसकी कोई साधारण वजह नहीं थी।
   d. Intended Interpretation: The individual recalls an occasion when they saw a light or lights that didn't seem to have a typical or everyday source, such as light bulbs or the sun.
   e. Coding Notes: Examples include but are not limited to a light around another person (an aura), a light in the sky or in nature (e.g., a UFO), spheres of light floating around ("orbs"), the light at the end of the tunnel associated with NDEs. The subject may see it alone or with others. 'Seemed' is meant to indicate that the respondent should choose 'Yes' if the light *appeared* to have no ordinary cause, even if the respondent

believes, upon further reflection, that the appearance of the light can be explained scientifically or medically (e.g., visual cortex seizures), or in terms of ordinary phenomena (e.g., aircraft, optical illusions).

f. Sources: This item was inspired by items that referred to light or luminosity in the KS, SAE, and NDES.

g. Iteration Count & Extend of Change: 4 in English, 2 in Hindi. Minor change.

h. First Iteration: I have perceived light or lights for which there seemed to be no obvious cause. / Maine aisa prakash ya roshni dekhi hai jiska koi spashta kaaran nahi tha.

15. **Sounds (Voices).**

a. I have heard a voice when it did not seem like anyone was really there.

b. Asadharan roop se maine kisi ki awaj ya boli suni hai lekin wahan koi bhi aur nahi tha.

c. असाधारण रूप से मैंने किसी की आवाज़ या बोली सुनी है लेकिन वहाँ कोई भी और नहीं था।

d. Intended Interpretation: The individual recalls an occasion when they heard a voice even though no one was actually present.

e. Coding Notes: It should have the qualities of a regular voice whether they experience it as arising internally or externally. The subject may hear it alone or with others. We leave the appraisal of its source and significance to the appraisals.

f. Source: This item was inspired by items that referred to hearing things when no one was around in the AANEX, DES, KS, and PAGE-R.

g. Iteration Count & Extend of Change: 3 in English, 3 in Hindi. Major change.

h. First Iteration: I have heard noises, voices, or music for which there seemed to be no obvious cause. / Maine aisa awaj ya sangeet suna hai jiska koi spashta kaaran nahi dikh raha tha.

16. **Touch.**

a. I have felt a physical touch when it did not seem like anyone was really there.

b. Maine kisi aur dwara ek asadharan sparsh ka anubhav kiya hai jab mere aajubaaju koi bhi nahi tha.

c. मैंने किसी और द्वारा एक असाधारण स्पर्श का अनुभव किया है जब मेरे आजूबाजू कोई भी नहीं था।

d. Intended Interpretation: The individual recalls an occasion when they felt a touch that seemed to come from another person even though no one was actually present.

e. Coding Notes: It should have the qualities of a regular human touch regardless of whether they believe it was real or not. The subject may feel it alone or with others. If there is spiritual healing associated with the touch that is fine, but not sufficient.

f. Source: This item was inspired by items that referred to feeling touched when no one was around in the PAGE-R and SAE.

g. Iteration Count & Extend of Change: 3 in English, 2 in Hindi. Minor change.

h. First Iteration: I have felt a physical touch for which there seemed to be no obvious cause. / Mujhe aisa sparsh ka anubhav hua hai jiski koi sadharan vajah nahi thi.

17. **Faces.**

a. I have seen what seemed like a face in a natural or human-made object.

b. Maine kudrati athva manushya dwara banayi vastu mein chehre jaisa kuch dekha hai.

c. मैंने कुदरती अथवा मनुष्य द्वारा बनाई वस्तु में चेहरे जैसा कुछ देखा है।

d. Intended Interpretation: Seeing faces in everyday objects.

e. Coding Notes: Examples include seeing a face in a U.S. electrical socket, the face of Jesus in a tortilla, the face of Ganesh in a tree, etc. In psychology, this is referred to as pareidolia.

f. Source: This item was inspired by reports of people seeing sacred figures in everyday objects or natural formations.

g. Iteration Count & Extend of Change: 2 in English, 3 in Hindi. Minor change.

h. First Iteration: I have seen a face in a natural or human-made object. / Maine kudarati athva manav nirmit vastu mein ek chehra dekha hai.

18. **Paralysis.**
    a. I have been unable to move or communicate because my body, my limbs, or my voice was paralyzed.
    b. Muje anubhav hua hai jaise mein na toh bol pa raha tha aur na toh chal pa raha tha kyon ki mera shareer stabdha ho gaya tha ya mujhe lakva maar gaya tha.
    c. मुझे अनुभव हुआ है जैसे मैं न तो बोल पा रहा था और न तो चल पा रहा था क्योंकि मेरा शरीर स्तब्ध हो गया था या मुझे लकवा मार गया था।
    d. Intended Interpretation: An experience in which the individual is unable to move their entire body or a part thereof.
    e. Note: It can be due to various causes, such as accident, medical condition (e.g., seizure), sleep paralysis, or fright.
    f. Source: This item was inspired by a similar item in the PAGE-R.
    g. Iteration Count & Extend of Change: 2 in English, 1 in Hindi. Minor change.
    h. First Iteration: I have not been able to move or communicate because my body, my limbs, or my voice seemed paralyzed. / Muje anubhav hua hai jaise mein na toh bol pa raha tha aur na toh chal pa raha tha kyon ki mera shareer stabdha ho gaya tha ya mujhe lakva maar gaya tha.
19. **Pain.**
    a. I have had an experience of physical pain that stood out from all other such experiences.
    b. Maine aisa shaaririk dard mehsus kiya hai jo baki aise anubhavo se alag raha ho.
    c. मैंने ऐसा शारीरिक दर्द महसूस किया है जो बाकि ऐसे अनुभवों से अलग रहा हो।
    d. Intended Interpretation: A strongly unpleasant or agonizing physical sensation (Adapted from OED, 3a).
    e. Source: This item is included as a negative experience that may be moderated by appraisals.
    f. Iteration Count & Extend of Change: 3 in English, 3 in Hindi. Major change.
    g. First iteration: I have had an experience of pain that stood out from all other such experiences. / Maine aisa dard mehsus kiya hai jo baki aise anubhavo se alag raha ho.

SENSE OF SELF

20. **Absorbed**.
    a. I have had an experience in which I was completely absorbed in what I was doing and unaware of the passage of time.
    b. Mujhe aisa anubhav hua jaise mei kisi karya mei puri tarah se jud gaya tha/gayi thi ki mujhe samay beetne ka ehsas hi nahi hua.
    c. मुझे ऐसा अनुभव हुआ जैसे मैं किसी कार्य में पूरी तरह से जुड़ गया था / गयी थी की मुझे समय बीतने का एहसास ही नहीं हुआ।
    d. Intended Interpretation: Experiences in which a person is so engrossed in an activity that they are completely unaware of what else is going on around them. They are completely focused on what they are doing, and they may mention being unaware of time passing.
    e. Coding Notes: It may be experienced as a "flow state." Yaden et al. [23] consider absorption as the most common (routine, ordinary) form of self-transcendent experience (STE), which they define as "transient mental states marked by decreased self-salience and increased feelings of connectedness ['with other people or one's surroundings']." Because flow states are often associated with practiced activities, we expect people may indicate a type of activity in which they routinely are absorbed rather than a specific instance of absorption.
    f. Related items: **Absorption** can be linked to **Automaticity** in cases where (e.g.) absorption in thoughts or a conversation results in performing actions automatically.
    g. Source: This item was inspired by items that referred to being absorbed or otherwise caught up in various kinds of thoughts or activities in the CEQ, DES, and TAS, as well as by its association with visualization and meditation practices.
    h. Iteration Count & Extend of Change: 1 in English, 1 in Hindi. No change.

21. **OBE.**
   a. I have had an experience in which it seemed as if I left my physical body.
   b. Muje aisa anubhav hua hai jahan muje laga ki mei apne bhautik sharir se juda ho chuka hun.
   c. मुझे ऐसा अनुभव हुआ है जहाँ मुझे लगा की में अपने भौतिक शरीर से जुदा हो चुका हूँ।
   d. Intended Interpretation: Experiences in which the person feels that they are leaving, are rising out of, or otherwise exiting their body and/or experiences in which they feel they are outside their body based on various cues including looking at their body from the outside, seeing things they normally would not see if they were in their body.
   e. Coding Notes: This includes experiences in which 'they' (e.g., their mind, soul, spirit) travel someplace in this or another world in a way that is not possible with their body. Dreams in which any of this occurs are included. It can also occur in the context of sleep paralysis. There has to be an implicit sense of self -- an "I" -- apart from the body; the "I" can't just dissolve.
   f. Source: This item was inspired by similar items in the AEI, CEQ, KS, TAS, PAGE-R, SAE, and NDES.
   g. Iteration Count & Extend of Change: 1 in English, 1 in Hindi. No change.
22. **Diminished Self.**
   a. I have felt small or insignificant relative to something vast or powerful.
   b. Muje aisa anubhav hua hai jaise mai kuch vishal ya bhavya ke samne kuch bhi nahi hun.
   c. मुझे ऐसा अनुभव हुआ है जैसे मैं कुछ विशाल या भव्य के सामने कुछ भी नहीं हूँ।
   d. Intended Interpretation: The feeling that one's self is diminished in the face of something with immense scope or power.
   e. Coding Notes: The intended interpretation thus includes vastness or power in terms of physical size, social status, loud sounds, the ground shaking, or symbolic vastness. The experience can be positive or negative. It may involve a positive sense that one is a small part of something greater, or a negative sense that one is small or inferior relative to something larger. This item does not require the loss of a sense of self, only the sense of diminished self, i.e., feeling small, insignificant, or, in the Hindi translation, as if one is nothing, in relation to something vast or powerful.
   f. Source: This item was inspired by the literature on awe, its association with experiences of transcendence, and items related to the feeling of unity with a larger whole in the MEQ30.
   g. Iteration Count & Extend of Change: 3 in English, 4 in Hindi. Minor change.
   h. First Iteration:I have felt small or insignificant in relationship to something vast and powerful. / Mujhe kisi vishal ya shaktishali taakat ke saamne nyunata/tuchhta ka anubhav hua hai.
23. **Automaticity.**
   a. I have had an experience in which it seemed like my body was performing actions outside my control (such as moving, speaking, or writing).
   b. Muje aisa anubhav hua jaise mera sharir, meri awaz ya meri likhai mere niyantran ke bahar kuch kam kar rahe the.
   c. मुझे ऐसा अनुभव हुआ जैसे मेरा शरीर, मेरी आवाज़ या मेरी लिखाई मेरे नियंत्रण के बहार कुछ काम कर रहे थे।
   d. Intended Interpretation: Experiences in which the whole body or a part of the person's body seems to be acting involuntarily or of its own accord.
   e. Coding Notes: The body has to be doing something (i.e., moving, speaking, writing) that the subject feels they are not intending or controlling. In other words, it is "not me" that is making my body move. The body should not be paralyzed or immobile (that’s our Paralysis item). The person might collapse under a perceived pressure (e.g., catalepsy, falling in the Spirit), get up and do things while asleep (sleep walking), feel as if their voice or speech was not their own (speaking in tongues), or that their hand is writing things independent of 'them' (automatic writing). It may occur in hindsight, after realizing that they have no memory of doing something, such as driving somewhere. Automaticity suggests the possibility that some "other" agent is present who is in control, but the extent to which the "other" is expressed is left open and claims regarding the presence of another agent are left to the appraisals. Automaticity in the context of drunkenness is OK. Ordinary fainting or collapsing from exhaustion does not count.

f. Sources: This item was inspired by items that referred to speaking in tongues (AEI), feeling as if singing or writing is directed by someone outside the self (CEQ), bodily movements that are involuntary or seemingly controlled by others (AANEX, O-Life, PAGE-R), one's body not seeming to be one's own (DES, O-LIFE).
g. Iteration Count & Extend of Change: 3 in English, 3 in Hindi. Minor change.
h. First Iteration: I have had an experience in which it seemed like my body, my voice, or my writing was operating outside of my own control. / Muje aisa anubhav hua jaise mera sharir, meri awaz ya meri likhai mere niyantran ke bahar ho chuke hai

PRESENCE-RELATED

24. **Presence (non-ordinary).**

a. I have sensed the presence of what seemed to be non-ordinary forces or beings.
b. Maine asadharan shaktiyo ya vastuo ki upasthiti ko mehsus kiya hai.
c. मैंने असाधारण शक्तिओं या वस्तुओ कि उपस्थिति को महसूस किया है।
d. Intended Interpretation: A sense of a nonphysical "other," whether perceived as an agent (e.g., deity, spirit, ancestor, dead person, alien, or alter personality) or a more amorphous power, force, or energy, that seems present based on some sort of perceived internal or external cues or messages.
e. Coding Notes: Although alter personalities can be construed as having a body, they are included as long as they seem to have a "mind of their own." Beings, such as tulpas [24], that have been cultivated are fine. The perceived presence can be positive or negative, sought or unsought, welcome or unwelcome. The nonphysical power or agent can be disembodied or thought to manifest through a living being or material object. The power or being may simply seem to be present; whether the subject actually thinks it is present will come out in the appraisals.
f. Differential diagnosis: Signs or messages without the sense of an "other" being present fall under our **Messages** item.
g. Related Items: This is the most inclusive of our Presence-related items. The others are all more specific.
h. Sources: This item was inspired by items that referred to ghosts, apparitions, elves, and aliens (AEI), God's presence (DSES), evil presences (O-LIFE), invisible forces, energies, or entities (PAGE-R), and mystical beings or presences (NDES).
i. Iteration Count & Extend of Change: 1 in English, 2 in Hindi. Minor change.
j. First Iteration, if different: Maine asadharan shaktiyo ya vastuo ki upasthiti ka anubhav kiya hai

25. **Guidance.**

a. I have had an experience of being guided or influenced by what seemed to be a non-ordinary power or being.
b. Mujhe aisa anubhav hua jaise mujhe koi anya takat nirdesh de rahi hai ya guide kar rahi hai.
c. मुझे ऐसा अनुभव हुआ जैसे मुझे कोई अन्य ताकत निर्देश दे रही है या गाइड कर रही है।
d. Intended Interpretation: A sense of a nonphysical "other," whether perceived as an agent (e.g., deity, spirit, ancestor, dead person, or alter personality) or a more amorphous power, force, or energy, that conveys a sense of what the individual should do through some sort of perceived internal or external cues or messages or by taking charge of the person's body.
e. Coding Notes: The perceived guidance or influence can be positive or negative, sought or unsought, welcome or unwelcome. The nonphysical power or agent can be immaterial or thought to manifest through a living being or material object. The power or being may simply *seem* to be present; whether the subject actually thinks it is present will come out in the appraisals. Note: Alter personalities are included as long as they seem to have a "mind of their own" and offer guidance. Ditto for Jungian type inner guides generated through active imagination and novelists whose characters come to life and tell them what to write.
f. Differential diagnosis: Signs or messages without the sense of an "other" fall under our **Messages** item. The power or being should not simply be present as in our **Presence (non-ordinary)** item, but must be perceived to communicate in some way. The power or being should not totally 'take over' or replace the person's sense of self or identity.
g. Related Items: There is potential overlap between this item and other Presence-related items in so far as the "other" is viewed as guiding.

h. Source: This item was inspired by an item that referred to feeling guided by God in the DSES.
i. Iteration Count & Extend of Change: 3 in English, 2 in Hindi. Major change.
j. First Iteration: I have had an experience of being sustained or guided by a power other than myself. / Muje aisa anubhav hua ki koi anya takat nirdesh ya madad kar rahi hai.

26. **Places (animated)**.
   a. I have experienced a particular place that seemed to contain a non-ordinary presence or power.
   b. Mai aisi jagah par gaya hun jahan koi asadharan upasthiti ya taakat mahsoos ki hai aur is wajah se vah sthaan vishishta hai.
   c. मैं ऐसी जगह पर गया हूँ जहाँ कोई असाधारण उपस्थिति या ताकत महसूस की है और इस वजह से वह स्थान विशिष्ट है।
   d. Intended Interpretation: This item is derived from the concept of animism, which is the perception that any thing—animals, plants, rocks, rivers, weather systems, human handiwork and perhaps even words—may contain a nonordinary presence or power that animates it. We are asking whether people have experienced a particular place (or type of place) in the natural world or in the built environment that seemed to contain a nonordinary presence or power.
   e. Coding Notes: Examples might include mountains, forests, the ocean, or other natural features that contain spirits; places such as Mt Shasta and Sedona that are considered spiritual vortices by New Age types; human built spaces, such as cathedrals, graveyards, or haunted houses. The power or being may simply *seem* to be present; whether the subject actually thinks it is present will come out in the appraisals.
   f. Differential Diagnosis: It contrasts with **Objects (animated),** which refers to nonordinary presences or powers in images, statues, or other physical objects rather than in places or spaces.
   g. Related items: There is potential overlap between this item and other Presence-related items in so far as the "other" is viewed as connected to a particular place in the natural world or built environment. This item overlaps with **Places (special)** in so far as the presence of an "other" makes the place special.
   h. Source: This item was inspired by indigenous traditions that view spirits and other supernatural entities as present in places in the natural world.
   i. Iteration Count & Extend of Change: 1 in English, 3 in Hindi. Minor change.
   j. First Iteration, if different: Maine kisi ek jagah par koi asadharan aakarshan, shakti ya astitva mahsoos kiya hai.

27. **Objects (animated)**
   a. I have seen and/or interacted with images, statues, or other physical objects that seemed to contain a non-ordinary power or being.
   b. Mai aisi pratima, murti, ya anya bhautik vastuon ke sampark me aayi/aaya hu jo jaagruk ya zinda prateet ho rahe the.
   c. मैं ऐसी प्रतिमा, मूर्ति, या अन्य भौतिक वस्तुओं के संपर्क में आया/आयी हु जो जागरुक या ज़िंदा प्रतीक हो रहे थे।
   d. Intended Interpretation: A sense of a nonphysical "other," whether perceived as an agent (e.g., deity, spirit, ancestor, dead person, or alter personality) or a more amorphous power, force, or energy, that seems to be present in an image, statue, or other physical object.
   e. Coding Notes: They may 'perceive' the presence of the other in the object based on some sort of perceived internal or external cues or messages and/or interact with the presence. The power or being may simply *seem* to be present; whether the subject actually thinks it is present will come out in the appraisals. Examples include Catholic Eucharistic wafers, Eastern Orthodox icons, and Hindu statues insofar as a deity is thought to be present in them.
   f. Related items: There is potential overlap between this item and other Presence-related items in so far as the 'presence' or 'power' is linked to a physical object. This item overlaps with **Devotion (Objects)** in so far as the object is thought to contain a nonordinary presence or power.
   g. Source: This item was inspired by traditions, such as Hinduism, Catholicism, and Eastern Orthodoxy, that view deities as present in objects.
   h. Iteration Count & Extend of Change: 2 in English, 1 in Hindi. Minor change.

i. First Iteration, if different: I have interacted with images, statues, or other physical objects that seemed to be conscious or alive.

PARANORMAL / ENHANCED ABILITIES

28. **Lucid Dreaming.**
   a. I have had the experience of being aware that I was dreaming while asleep.
   b. Swapna dekhte samay hi muje aisa anubhav hai jahan muje spashtarup se pata ho ki sachhai nahi lekin swapna dekh raha hun.
   c. स्वप्न देखते समय ही मुझे ऐसा अनुभव है जहाँ मुझे स्पस्टरूप से पता हो की सच्चाई नहीं लेकिन स्वप्न देख रहा हूँ।
   d. Intended Interpretation**:** While dreaming, the respondent is aware that they are dreaming. They may or may not feel that they can control their own actions or the events in the dream.
   e. Note: In the Hindi version, the emphasis was mistakenly left out of the item during validation. The consensus of our Hindi-speaking team members was that the emphasis would not substantially change the interpretation, aside from potentially improving clarity. The item performed well without the emphasis, but we recommend including the emphasis.
   f. Source: This item was inspired by an item that referred to the ability to control one's dreams in the AEI and traditions that cultivate this ability as a portal into "other worlds."
   g. Iteration Count & Extend of Change: 2 in English, 2 in Hindi. Minor change.
   h. First Iteration: I have had the experience of being aware that I was dreaming while asleep. / Swapna dekhte samay hi muje aisa anubhav hai jahan muje spashtarup se pata ho ki sachhai nahi lekin swapna dekh raha hun.

29. **Déjà vu.**
   a. I can recall one specific experience in which a new situation felt so familiar to me that it seemed as if I had been there before.
   b. Ek aisi vishista paristhiti jiska anubhav pahle kabhi nahi hua us paristhiti ka anubhav hote waqt aisa laga jaise aisa mere saath ho chuka hai.
   c. एक ऐसी विशिष्ट परिस्थिति जिसका अनुभव पहले कभी नहीं हुआ उस परिस्थिति का अनुभव होते वक्त ऐसा लगा जैसे ऐसा मेरे साथ हो चूका है।
   d. Intended Interpretation**:** A paradoxical feeling of having already experienced the present situation when it seems as if the situation couldn't have happened before. An illusory feeling of having previously experienced a present situation; recollecting a scene which is only now occurring for the first time (OED).
   e. Related item**:** Past Life and Déjà vu can overlap if the déjà vu experience involves a specific memory that is thought to be impossible.
   f. Source: This item was inspired by items in the AEI, DES, and PAGE-R.
   g. Iteration Count & Extend of Change: 3 in English, 3 in Hindi. Minor change.
   h. First Iteration: I have experienced a new situation that felt so familiar to me that it seemed as if I had been in it before. / Maine ek nayi paristhiti mei aisa anubhav kiya hai jaise ki vah parishiti ka anubhav muje pahle kabhi ho chuka hai.

30. **Past Life.**
   a. I have distinct memories that made it seem as if I had lived a past life in a different body.
   b. Mere paas spashta smritiyan hai jaise ki mei kisi anya sharir mei ek dusri jindagi bita chuka/chuki hun.
   c. मेरे पास स्पष्ट स्मृतियाँ है जैसे कि मैं किसी अन्य शरीर में एक दूसरी ज़िंदगी बिता चुका / चुकी हूँ।
   d. Intended Interpretation**:** Memories of events or actions that do not seem connected to 'this life' and from which people might infer that they had lived a life in another time.
   e. Coding Notes: This can include memories of events at which they seemed to be present that they could not have witnessed in this life and/or memories of how to do things (e.g., to compose or play music, or solve math problems) that it seemed like they could not have learned in this lifetime. The key is that the memories do not seem connected to this life; the memories could have arisen in a dream. 'Seemed' is meant to indicate

that the respondent should choose 'Yes' even if the respondent believes, upon further reflection, that they did not actually live a past life.

f. Differential diagnosis: The person may have a sense of deja vu (a sense of having been somewhere before) but they have to have a distinct memory of being there and some reason they could not have been there for it to qualify for this item.
g. Source: This item was inspired by items in the AEI and SAE.
h. Iteration Count & Extend of Change: 3 in English, 2 in Hindi. Minor change.
i. First Iteration: I have distinct memories that made it seem as if I had lived a different life, in a different time and place. / Mere paas spashta smritiyan hai jaise ki mei kisi anya jagah aur waqt par ek dusri jindagi bita chuka/chuki hun.

31. **ESP (events).**
   a. I have had an impression that a specific event was occurring at some distant location that later turned out to be true.
   b. Muje aisa khudse hi mahsoos hua ki koi vishista ghatna door ke sthan par ghatit ho rahi hai aur baad mei pata chala ki aisa sachmuch hua tha.
   c. मुझे ऐसा खुदसे ही महसूस हुआ कि कोई विशिष्ट घटना दूर के स्थान पर घटित हो रही है और बादमें पता चला की ऐसा सचमुच हुआ था।
   d. Intended Interpretation: This item refers to the reception of information (via a sudden feeling or other means that does not involve the five senses) regarding a specific event that is taking place at a distance and later is confirmed.
   e. Coding Notes: The subject should not have had prior knowledge of the event or, if they do (e.g., if they know a family member is likely to die soon), they don't know when it will occur. They should describe a specific event. The information can come in any mental state including a dream.
   f. Coding: Responses that were coded as "Not Understood" were experiences of precognition or being aware that something will happen before it happens, which may be immediate rather than distant (e.g., having a feeling that a truck is about to pull out in front of you). This interpretation was more common in the US than in India (see validation spreadsheets for both this item and the appraisals), so differences in frequency between these populations should be interpreted with this in mind. This item was adapted from the SAE (Irwin 2013) after testing various less specific ESP items.
   g. Source: This item was initially inspired by items referring to psychic experiences related to events occurring in distant locations in the AEI, PAGE-R, SAE, and NDES, but eventually based on an item in the SAE.
   h. Iteration Count & Extend of Change: 4 in English, 3 in Hindi. Major change.
   i. First Iteration: I have received or become aware of information, through what seemed to be non-ordinary means, about the past, future, or a distant location, that turned out to be true. / Muje asadharan roopse bhootkal, bhavishyakaal ya anya doorsthit sthaan ke bare mei jankari prapt hui jo vastav mei hakikat sabit hui.

32. **ESP (minds).**
   a. I have had the experience of thinking of a person I haven't heard from in ages, and later in the day they contacted me.
   b. Muje aisa anubhav hua hai jahan mei ek din aise vyakti ko yaad kar raha tha jisase maine bahut samay se mulakat nahi ki, usi vyakti se usi din mulakat ho gayi.
   c. मुझे ऐसा अनुभव हुआ है जहाँ मैं एक दिन ऐसे व्यक्ति को याद कर रहा था जिससे मैंने बहुत समय से मुलाकात नहीं की, उसी व्यक्ति से उसी दिन मुलाकात हो गयी।
   d. Intended Interpretation: On the same day that the respondent thought about someone they had not been in contact with for a while, that person contacted them.
   e. Note: This item was adapted from the SAE after testing various less specific ESP items.
   f. Source: This item was initially inspired by items referring to psychic experiences related to the minds of others in the AEI, ANNEX, CEQ, TAS, PAGE-R, and SAE, but eventually based on similar items in the CEQ and SAE.
   g. Iteration Count & Extend of Change: 6 in English, 5 in Hindi. Major change.

h. First Iteration: I have perceived, through what seemed to be non-ordinary means, the thoughts and feelings of other people. / Muje lagata hai ki mein anya logo ki bhavnao aur vicharo ko asadharan roop se pahchan leta hu.

SICKNESS / HEALTH

33. **Healing.**

a. I have experienced a sudden and unexpected recovery from an illness (physical, mental, or any other kind).
b. Maine sharirik, mansik ya koi anya prakar ki bimari se achanak aur ascharyajanak ilaaj (durusti) ka anubhav kiya hai jo baki aise anubhavo se alag tha.
c. मैंने शारीरिक, मानसिक या कोई अन्य प्रकार की बीमारी से अचानक और आश्चर्यजनक इलाज (दुरुस्ती) का अनुभव किया है जो बाकि ऐसे अनुभवों से अलग था।
d. Intended Interpretation: Any experience of recovery from an illness, whatever the nature or presumed cause, as long as the recovery is sudden and unexpected.
e. Source: This item was inspired by items that referred to healing via healing energy (AEI) and the power of the mind (SAE).
f. Iteration Count & Extend of Change: 6 in English, 5 in Hindi. Major change.
g. First Iteration: I have had an experience of healing or being healed that stood out from all other such experiences. / Mujhe aisa anubhav hua hai ki mere maansik ya shaaririk swaasthya mei ek alag hi sudhaar hua hai jo baki sadharan aise anubhavo se bahut alag tha.

34. **Near Death**:

a. I have had an experience in which I felt as if I was about to die.
b. Mujhe aisa anubhav hua hai jahan mujhe laga ki ab kuch hi kshan me mai marne wala hun.
c. मुझे ऐसा अनुभव हुआ है जहाँ मुझे लगा की अब कुछ ही क्षणमें मैं मरने वाला हूँ।
d. Intended Interpretation: An experience in which the person feels as if they are about to die. The feeling must be present at the time the event occurred (or immediately thereafter).
e. Coding Notes: Examples include life-threatening events such as illnesses, accidents, or natural disasters. Experiences retrospectively appraised as NDEs do not count if the individual did not feel they were about to die at the time the event occurred or immediately thereafter.
f. Source: This literal “near death” item was added because “close calls” may elicit spiritual appraisals.
g. Iteration Count & Extend of Change: 1 in English, 2 in Hindi. Minor change.
h. First Iteration, if different: Mujhe ek aisa anubhav hua hai jismei mujhe laga jaise mei marne wala hun.

MEANING

35. **Coincidences.**

a. I have experienced a coincidence that seemed meaningful.
b. Maine ittefaq - yani do ghatnae jinka aapas mei sambandh na ho lekin ve ek saath ho jaay - aisa anubhav kiya hai jo bahut arthapoorna raha ho.
c. मैंने इत्तेफ़ाक़ - यानि दो घटनाएँ जिनका आपस में सम्बन्ध न हो लेकिन वे एक साथ हो जाय - ऐसा अनुभव किया है जो बहुत अर्थपूर्ण रहा हो।
d. Intended Interpretation: “A notable concurrence of events or circumstances having no apparent causal connection.” (OED, 4).
e. Coding Notes: Responses often involve a chance meeting with another person, often an old friend. Note: responses occasionally include relatively isolated events that go on to make an impact in one’s life, e.g. meeting someone who becomes a good friend.
f. Source: This item was inspired by items that could be construed as coincidences in the AANEX, O-Life, PAGE-R, and SAE, as well as by traditions that claim that there are no coincidences because nothing happens by chance.
g. Iteration Count & Extend of Change: 1 in English, 3 in Hindi. Minor change.

   h. First Iteration: Maine ek aise ittefaq/saiyog ka anubhav kiya hai jo mujhe bahut saarthak/arthpoorn laga.

36. **Messages.**
   a. I have experienced or felt things in the world around me that seemed to contain messages or hints.
   b. Muje apne aas-paas ki duniya mei aisa anubhav hua hai jaise ki inmei kuch sandesh ya sanket chupe hai.
   c. मुझे अपने आस-पास की दुनिया में ऐसा अनुभव हुआ है जैसे कि इनमें कुछ सन्देश या संकेत छुपे है।
   d. Intended Interpretation: The subject perceives things around them as conveying special information (messages or hints) directed to them that others might not recognize as such.
   e. Note: The source of the message does not need to be specified; there does not have to be a "sender."
   f. Source: This item was adapted from an item in the AANEX that referred to messages or hints.
   g. Iteration Count & Extend of Change: 1 in English, 1 in Hindi. No change.

37. **Deep Insight.**
   a. I have had a sudden, deep insight in which the nature of reality, the functioning of society, or other fundamental issues seemed clear.
   b. Mujhe aisa anubhav hua hai jaise maine achanak hi vaastavikta ke gahre rahasyo ko yatharth roopse samajh liya hai.
   c. मुझे ऐसा अनुभव हुआ है जैसे मैंने अचानक ही वास्तविकता के गहरे रहस्यों को यथार्थ रूपसे समज लिया है।
   d. Intended Interpretation: A sudden deep understanding of a fundamental issue. An 'aha' moment.
   e. Coding Notes: The "insight" can be of any fundamental issue, e.g., the nature of reality, the meaning of life, human nature, society, etc. It can involve a sense of "enlightenment" or "revelation." The insight has to be deep and involve a sudden sense of clarity (an 'aha' moment).
   f. Related item**:** This may overlap with Meaning in Life.
   g. Source: This item was inspired by items that referred to insights or sudden revelations in the AANEX, MS, and MEQ30.
   h. Iteration Count & Extend of Change: 2 in English, 3 in Hindi. Minor change.
   i. First Iteration: I have experienced a sudden deep insight in which the nature of reality, the functioning of society, or other fundamental issues suddenly seemed clear. / Mujhe aisa anubhav hua hai jaise maine jeevan ki vaastavikta ko yatharth roopse samajh liya hai ya gahrai se pahchan liya hai..

38. **Meaning in Life.**
   a. I have had an experience in which the meaning and purpose of my life suddenly seemed clear.
   b. Mujhe aisa anubhav hua hai jaise mere jeevan ka matlab ya uddeshya achaanak hi spashta ho gaya hai.
   c. मुझे ऐसा अनुभव हुआ है जैसे मेरे जीवन का मतलब या उद्देश्य अचानक ही स्पष्ट हो गया है।
   d. Intended Interpretation: A sudden experience of clarity with respect to the meaning or purpose of one's life. The experience may involve a sense of direction, calling, spiritual awakening, vocation or duty.
   e. Related Items: This may overlap with **Deep Insight**.
   f. Source: This item was inspired by an item in the AANEX that referred to one's mission or duty being revealed.
   g. Iteration Count & Extend of Change: 1 in English, 1 in Hindi. No change.

Follow-up Items

Mental State: When you had the experience, were you … (Select the most important)

- Using drugs or alcohol
- Affected by mental or physical illness
- Falling asleep, waking up, or exhausted
- Asleep (dreaming)
- None of the above

Intended Interpretation: This item asks about the mental state or context in which the experience occurred.

- Using drugs or alcohol: The experience occurred while they were under the influence of a substance that has a physiological effect when ingested or otherwise introduced into the body (adapted from Oxford Online). Technically alcohol is a drug.
- Affected by mental or physical illness: The experience occurred during a period of sickness that potentially affected their body or mind (adapted from Oxford Online).
- Falling asleep, waking up, or exhausted: The experience occurred in a state immediately preceding waking up (hypnopompic), immediately before falling asleep (hypnagogic), or physically or mentally exhausted.
- Asleep (dreaming): The experience occurred while asleep, typically while dreaming.

Note: We tested two versions of a practice-related response option, i.e., “Engaging in practices related to the experience” and “Doing something intended to bring about such an experience.” Neither option was consistently understood in the way we intended.

First Iteration: In what context did this experience occur? (Select the most important.) (Awake, in association with “mind altering” practices; Awake, in association with drugs or alcohol; Due to mental or physical illness; Falling asleep, waking up, or exhausted; Asleep (dreaming); None of the above (awake, normal state))

Impact: Overall, how much of an impact has this experience had on your life?

●○○ Little or no impact
●●○ Some impact
●●● Major impact

Intended Interpretation: This item asks about the extent to which the experience had an effect on the person’s life mentally or physically, regardless of whether they view the effect as positive, negative, or mixed. The effect can be on their beliefs (worldview), behavior, or life course.

- Little or no impact -- the experience had negligible effect on their life both at the time it occurred and thereafter. Reasons why they selected this answer might include: “It didn’t change anything.” “Because I only remember the experience if something triggers my memory.” “It’s something I’m thankful for, but it doesn’t change much.”
- Some impact -- the experience had a noticeable, but not particularly significant, effect on their life at the time it occurred and/or thereafter. Explanations of “some impact” usually include a “but” or its equivalent. Examples include: “It wasn’t something that changed my life … It did cause me to become more interested in nature, however.” “It wasn’t life altering [but] it definitely made me more aware of my surroundings and my friendships.” “It didn’t affect me as much as other things in my life [but] I still question it and it was a very odd experience.” “Because it is only part of my life, it did not define everything.” “[Lucid dreams] made going to bed more exciting, but didn’t change the realities of my life.”
- Major impact - the experience changes their life (mentally or physically) in a very significant way at the time it occurred and/or subsequently. Examples of reasons for selecting this include: “It affects every aspect of my life.” “It was a turning point in my life.” “There was no going back to the way my life had been before.” “To pick up and move across the country [leaving kids and family] was a huge impact.”

First iteration: Overall, how significant has this experience been for you? (Not at all significant; Somewhat significant; Very significant)

Life Effect: Overall, has the **lasting** effect of this experience, on your life or beliefs, been more positive or negative?

- Very positive effect
- Somewhat positive effect
- Neutral or no effect
- Somewhat negative effect
- Very negative effect

Intended Interpretation: This item asks them to evaluate the lasting effects of the experience on their beliefs (worldview), behavior, and/or life course and assess whether the overall effect was positive, negative, or neutral. The effects are lasting if they are long term and continue in some fashion into the present.

- Very positive effect - the experience led to a very good or highly desirable outcome in some aspect of their life that has lasted to the present. For example: "After that experience [of being saved from an accident via Guidance] I was drastically changed ["my belief in God increased and I felt a lot of self-confidence in my life"].
- Somewhat positive effect - (1) on balance, the experience led to an outcome that was more positive than negative (more good than bad, more desired than not) or (2) the experience had an outcome that was good or desired, but the effects were somewhat attenuated over time (i.e., only somewhat lasting). For example: "This was a positive experience [ESP distant] but it did not have a lot of impact."
- Neutral or no effect - (1) on balance, the experience had a lasting effect that was equally positive and negative (equally good and bad, equally desired and not) or (2) the experience had little lasting effect one way or another. For example: "Because I knew that it [Lucid Dream] was a dream and what I was thinking was just a story. … As soon as I wake up in the morning, I get back to my routine job." "It was a weird but harmless experience." "It [a Loss] had both positive and negative effects, obviously, so they cancel each other out."
- Somewhat negative effect - (1) on balance, the experience led to an outcome that was more negative than positive (more bad than good, more undesirable than desired) or (2) the experience had an outcome that was bad or not desirable, but the effects were intermittent or somewhat attenuated over time (i.e., only somewhat lasting). For example: "It had some negative impact for a few days [but] after some time I came out of this despair." "I do not have these experiences [ESP distant] frequently and their impact [negative effect] is also only somewhat."
- Very negative effect - the experience led to a very bad or highly undesirable outcome in some aspect of their life that has lasted to the present. For example: "Because this experience [of despair] troubles me everyday and it has influenced me very negatively." "Because [of being awakened by a feeling of being pushed [Touch], I am now scared of sleeping alone."

First iteration. Has this experience had a lasting effect on your life or beliefs? (+2 Very positive effect; +1 Somewhat positive effect; 0 Little or no effect, positive or negative; -1 Somewhat negative effect; -2 Very negative effect)

Category (R/S): Do you consider this experience spiritual or religious?

- Yes
- No

Intended Interpretation: Because we want to know how respondents categorize their experience, we accept whatever answer they give us. In the Hindi version, the key terms are *adhyatmik* and *dharmik*. U.S. respondents' responses to the item can be interpreted in light of studies of the range of meanings among different U.S. populations [25–27].

Science: Do you think science can explain how this experience happened?

- Yes, science can or will be able to explain it.
- No, something More is involved.

Intended Interpretation: This item asks respondents to assess whether or they think science can or will be able to account for their experience. Participants' understandings of science may vary. Science can be understood generally or in terms of a

particular branch that relates to the participant's experience. Although the reference to "More" hearkens back to William James and was interpreted by most as a reference to spiritual or supernatural powers, we accepted any interpretation of "More" that went beyond science as the respondent understood it.

- Yes, science can or will be able to explain it. A "yes" response means that the respondent thinks a scientific explanation can or will be able to account for an experience.
- No, something More is involved. The "More" could be conceived, for example, as supernatural, spiritual, magical, occult, and/or paranormal. Their affirmation of some sort of nonscientific explanation should be evident either in their explanation of why they chose this response, their description of their experience, or their answers to other follow-up items.
- Note: Because some people believe that spiritual agents are "ultimate causes" and that such agents can and do act through natural (i.e., scientifically) explainable processes, a "yes" answer to this item is not necessarily incompatible with viewing the experience as spiritual or religious or even with thinking that a spiritual agent was ultimately responsible for it.

Reason: Why do you think it happened to you? (Select the closest answer.)

- To offer me a sign or message
- To reward or punish me for my actions
- Due to destiny/fate
- None of the above (may include chance/probability)

Intended Interpretation. This item is intended to capture respondents' efforts to make meaning of their experiences regardless of whether they considered themselves or the experience as religious or spiritual. They can have the feeling that it happened for a reason regardless of whether they consider the experience religious or spiritual and without believing or implying that there was an agent or force involved.

- To offer me a sign or message: The experience happened in order to convey something to them. Although the response implies that "someone" offered a sign or message, they do not need to specify -- or even assume -- that there was some sort of causal agent involved. The contents conveyed can be life changing or trivial. The information conveyed can be helpful or harmful, welcome or unwelcome. The nature of the information will vary depending on the situation. For example, it could contain useful information (e.g., where they can find a parking space), guidance (e.g., regarding a decision), or a warning (e.g., you need to change your life).
- To reward or punish me for my actions: They feel that the experience happened because they did something that has been judged good or bad. Although the response implies that "someone" judged their action good or bad, they do not need to specify -- or even believe -- that there was some sort of causal agent involved.
- Due to destiny/fate: They feel that the experience was "meant to happen" for some reason. Although the response implies that "someone" meant it to happen, they do not need to specify -- or even assume -- that there was some sort of causal agent involved.
- None of the above (may include chance/probability): Any answer that does not fit with the responses above is acceptable here. We explicitly mentioned chance and probability to indicate that they should select this option if they thought the experience occurred for "no reason." We accepted "bad luck" -- as in "it was just bad luck" as a synonym for chance/probability.

Agent: Who, if anyone, caused you to experience this? (Select the most important.)

- God or gods
- Other spiritual beings or forces (including the dead)
- None of the above

Intended Interpretation: This item was intended to determine whether respondents thought a nonphysical agent caused the experience. Like the Reason item, the Agent item does not distinguish between proximate causes (mechanisms directly

underlying the experience) and ultimate causes (evolutionary or historical explanations) and thus leaves open how the nonphysical agent is thought to have intervened to have caused the experience (i.e., through natural or supernatural means).

- God or gods includes any god, including any human if the human is thought to be a deity (e.g. Jesus, Krishna or other avatar).
- Other spiritual beings or forces (including the dead) is an expansive catch-all for spiritual beings or forces with presumed agent-like powers that are not viewed as deities. The agents may be viewed as benevolent or malevolent. Examples include angels, devils, demons, jinn, and fairies. Humans, animals, or aliens with presumed spiritual powers are also acceptable, e.g., gurus, saints, witches, and spirit animals. We added "including the dead," to capture ghosts, ancestors, and others who have died. We also included "spiritual forces" to capture more vaguely conceived spiritual agents, such as the Universe, the Source, or Fate.

Coding Notes: Some religious people answered the Reason question in light of proximate causes, but, when given these response options for the Agent question, shifted their focus to ultimate causes. If a respondent said "Yes, science can explain their experience" and selected either "God or gods" or "Other spiritual beings" in response to the Agent item, we coded their response as "understood" if they explained their Agent response is ultimate terms (e.g. God is the [ultimate] cause of everything) or gave evidence of their belief in spiritual agents (usually God) in their answers to the other items, including the demographics.

Version History. Descended from *Cause*: What do you believe caused this experience? Select the primary cause(s). (God, gods, or other deities; Other extraordinary or supernatural beings; Extraordinary or supernatural forces, powers, or abilities, including your own; Normal mental or physical processes; Drugs and/or alcohol; Mental or physical illness)

Hindi Follow-up Items (Roman and Devanagari)

Mental State: Jab aapko yah anubhav hua tab aap...

- Sharaab ya anya padarth ke nashe mei the.
- Mansik ya sharirik roop se bimar the.
- Thake hue, neend mei jate hue ya neend se jaag rahe the.
- Sapne mei the.
- In mei se koi bhi nahi.

जब आपको यह अनुभव हुआ तब आप...

- शराब या अन्य पदार्थ के नशे में थे
- मानसिक या शारीरिक रूप से बीमार थे
- थके हुए, नींद में जाते हुए या नींद से जागते हुए थे
- सपने में थे
- इन में से कोई भी नहीं

Impact: Sampoorna roop mei, is anubhav ka aapke jeevan par kitna **gahra asar** pada hai?

●○○ Koi asar nahi ya mamooli asar
●●○ Thoda bahut asar
●●● Bahut gahra asar

सम्पूर्ण रूप में, इस अनुभव का आपके जीवन पर कितना **गहरा असर** रहा है?

●○○ कोई नहीं या मामूली असर

●●○ थोड़ा बहुत असर
●●● बहुत गहरा असर

Life Effect:

Sampoorna roop mei—matlab **gahra aur sthayiroop se**—is anubhav ka aapke jeevan ya soch par kaisa prabhav raha hai - sakaratmak ya nakaratmak?

+2 bahut sakaratmak prabhav
+1 thoda bahut sakaratmak prabhav
0 koi prabhav nahi ya tatastha prabhav
-1 thoda bahut nakaratmak prabhav
-2 bahut nakaratmak prabhav

संपूर्ण रूप में—मतलब **गहरा या स्थायीरूप से**—इस अनुभव का आपके जीवन या सोच पर कैसा प्रभाव रहा है - सकारात्मक या नकारात्मक

+2 बहुत सकारात्मक प्रभाव
+1 थोड़ा बहुत सकारात्मक प्रभाव
0 कोई प्रभाव नहीं या तटस्थ प्रभाव
-1 थोड़ा बहुत नकारात्मक प्रभाव
-2 बहुत नकारात्मक प्रभाव

Category (R/S):

Kya aap is anubhav ko dharmik ya adhyatmik samajte hai?

- Haan
- Na

क्या आप इस अनुभव को धार्मिक या आध्यात्मिक समझते है?

- हाँ
- ना

Science:

Kya vigyaan samjha sakta hai ki yah anubhav kaise hua?

- Haan, vigyaan aaj ya bhavishya mei samjha sakta hai
- Nahi, vigyaan ke pare kuch aur badi baat hai

क्या विज्ञान समझा सकता है कि यह अनुभव कैसे हुआ?

- हाँ, विज्ञान आज या भविष्यमें समझा सकता है
- नहीं, विज्ञान के परे कुछ और बड़ी बात है

Reason:

Yah anubhav aapke saath kyun hua?

- Mujhe sanket ya sandesh dene ke liye
- Mere acche ya bure karmo ke phal-swarup
- Niyati ya bhagya ke karan
- Inmei se koi bhi nahi

यह अनुभव आपके साथ क्यों हुआ?

- मुझे संकेत या संदेश देने के लिए
- अच्छे या बुरे कर्म के फल के कारन
- नियती या भाग्य के कारण
- इनमें से कोई भी नहीं

Agent:

Yah anubhav jo hua vah kiske karan hua?

- Parameshwar / khuda / devata.
- Anya alaukik jeev ya shaktiyan.
- Inme se koi nahi.

यह अनुभव जो हुआ वह किस के कारण हुआ?

- परमेश्वर / खुदा / देवता
- अन्य अलौकिक जीव या शक्तियाँ
- इन में से कोई भी नहीं

References

1. Gallagher C, Kumar VK, Pekala RJ. The Anomalous Experiences Inventory: Reliability and validity. J Parapsychol. 1994;58:402–28.

2. Brett CMC, Peters EP, Johns LC, Tabraham P, Valmaggia LR, McGuire P. Appraisals of Anomalous Experiences Interview (AANEX): A multidimensional measure of psychological responses to anomalies associated with psychosis. Br J Psychiatry Suppl. 2007;51:s23-30.

3. Drinkwater K, Denovan A, Dagnall N, Parker A. The Australian Sheep-Goat Scale: An evaluation of factor structure and convergent validity. Front Psychol. 2018;9:1594.

4. Thalbourne MA, Delin PS. A new instrument for measuring the sheep-goat variable: Its psychometric properties and factor structure. J Soc Psych Res. 1993;59(832):172–86.

5. Merckelbach H, Horselenberg R, Muris P. The Creative Experiences Questionnaire (CEQ): A brief self-report measure of fantasy proneness. Personal Individ Differ. 2001 Oct;31(6):987–95.

6. Underwood LG, Teresi JA. The Daily Spiritual Experience Scale: Development, theoretical description, reliability, exploratory factor analysis, and preliminary construct validity using health-related data. Ann Behav Med. 2002;24:22–33.

7. Bernstein E, Putnam FW. Development, reliability, and validity of a dissociation scale. J Nerv Ment Dis. 1986;174:727–35.

8. Nour MM, Evans L, Nutt D, Carhart-Harris RL. Ego-dissolution and psychedelics: Validation of the Ego-Dissolution Inventory (EDI). Front Hum Neurosci. 2016;10:269.

9. Thalbourne MA. The Kundalini Scale and its relation to mystical and paranormal experience. In: Fallio VW, editor. New Developments in Consciousness Research. Nova Science Publishers; 2007. p. 119–31.

10. Barrett FS, Johnson MW, Griffiths RR. Validation of the revised Mystical Experience Questionnaire in experimental sessions with psilocybin. J Psychopharmacol. 2015 Nov;29:1182–90.

11. MacLean KA, Leoutsakos JMS. MS, Johnson MW, Griffiths RR. Factor analysis of the mystical experience questionnaire: A study of experiences occasioned by the hallucinogen psilocybin. J Sci Study Relig. 2012;51(4):721–37.

12. Hood RW. The construction and preliminary validation of a measure of reported mystical experience. J Sci Study Relig. 1975;14:29–41.

13. Greyson B. The Near-Death Experiences Scale: Construction, reliability, and validity. J Nerv Ment Dis. 1983;171(6):369–75.

14. Granqvist P, Hagekull B. Seeking security in the New Age: On attachment and emotional compensation. J Sci Study Relig. 2001 Sep;40(3):527–45.

15. Fach W, Atmanspacher H, Landolt K, Wyss T, Rössler W. A comparative study of exceptional experiences of clients seeking advice and of subjects in an ordinary population. Front Psychol. 2013;4:65.

16. Tobacyk JJ. A Revised Paranormal Belief Scale. Int J Transpers Stud. 2004 Jan 1;23(1):94–8.

17. Irwin HJ, Dagnall N, Drinkwater K. Parapsychological experience as anomalous experience plus paranormal attribution: A questionnaire based on a new approach to measurement. J Parapsychol. 2013;77(1):39–53.

18. Lange R, Ross RM, Dagnall N, Irwin HJ, Houran J, Drinkwater K. Anomalous experiences and paranormal attributions: Psychometric challenges in studying their measurement and relationship. Psychol Conscious Theory Res Pract. 2019 Dec;6(4):346–58.

19. Jamieson GA. The modified Tellegen Absorption Scale: A clearer window on the structure and meaning of absorption. Aust J Clin Exp Hypn. 2005;25.

20. Tellegen A, Atkinson G. Openness to absorbing and self-altering experiences ("absorption"), a trait related to hypnotic susceptibility. J Abnorm Psychol. 1974;83(3):268–77.

21. Lange R, Thalbourne MA, Houran J, Storm L. The Revised Transliminality Scale: Reliability and validity data from a Rasch top-down purification procedure. Conscious Cogn. 2000;9(4):591–617.

22. Mason O, Claridge G. The Oxford-Liverpool Inventory of Feelings and Experiences (O-LIFE): further description and extended norms. Schizophr Res. 2006;82(2–3):203–11.

23. Yaden DB, Haidt J, Hood RW, Vago DR, Newberg AB. The varieties of self-transcendent experience. Rev Gen Psychol. 2017;21(2):143–60.

24. Veissière SPL. Varieties of tulpa experiences: The hypnotic nature of human sociality, personhood, and interphenomenality. In: Raz A, Lifshitz M, editors. Hypnosis and meditation: Towards an integrative science of conscious planes. Oxford : New York: Oxford University Press; 2016. p. 55–74.

25. Altmeyer S, Klein C, Keller B, Silver CF, Hood RW, Streib H. Subjective definitions of spirituality and religion: An exploratory study in Germany and the US. Int J Corpus Linguist. 2015 Dec 31;20(4):526–52.

26. Altmeyer S, Klein C. "Spirituality" and "religion"—Corpus analysis of subjective definitions in the questionnaire. In: Streib H, Hood, RW, editors. Semantics and psychology of spirituality [Internet]. Cham: Springer International Publishing; 2016 [cited 2021 May 11]. p. 105–23. Available from: http://link.springer.com/10.1007/978-3-319-21245-6_8

27. Streib H, Klein C. Religion and spirituality. In: Stausberg M, Engler S, editors. The Oxford handbook of the study of religion. New York, NY: Oxford University Press; 2016. p. 73–83. (Oxford handbooks in religion and theology).
