# Supplementary material for: The Inventory of Nonordinary Experiences (INOE): Evidence of validity in the United States and India
Source: PLoS One. 2023 Jul 26;18(7):e0287780. doi: 10.1371/journal.pone.0287780 (PMC10370766; doi:10.1371/journal.pone.0287780)
Supplement: S2 Appendix — (PDF) [file pone.0287780.s002.pdf]

S2 Appendix
Unvalidated Items

Items are divided into two sections based on whether validation was attempted. Sources that inspired items are indicated with abbreviations where relevant.

AEI	The Anomalous Experience Inventory [1].
AANEX	The Appraisals of Anomalous Experiences Interview Probes [2].
ASGS	The Australian Sheep-Goat Scale [3,4].
CEQ	The Creative Experiences Questionnaire [5].
DSES	The Daily Spiritual Experience Scale [6].
DES	The Dissociative Experiences Scale [7].
EDI	The Ego Dissolution Inventory [8].
KS	The Kundalini Scale [9].
MEQ	The Mystical Experience Questionnaire [10,11].
MS	The Mysticism Scale [12].
NDES	The Near-Death Experience Scale [13].
NAOS	The New Age Orientation Scale [14].
PAGE-R	The Questionnaire for Assessing the Phenomenology of Exceptional Experiences [15].
RPBS	The Revised Paranormal Belief Scale [16].
SAE	The Survey of Anomalous Experiences [17,18].
TAS	The Tellegen Absorption Scale [19,20].
TS	The Transliminality Scale [21].
O-LIFE	The "Unusual Experience" Section of the Oxford-Liverpool Inventory of Feelings and Experiences [22].

Validation Attempted

The items we did attempt to validate are divided into four subsections: rare items associated with mystical experiences, other relatively rare items, items we were unable to ground, and items whose validity we questioned.

Rare items associated with mystical experience that failed to validate in the general population in one or both countries.

The item wording is given for the first iteration in the validation process. For the wording of subsequent iterations, please contact the authors.

1. **Connectedness (All).**
    a. I have had an experience in which I lost a sense of my individual identity and felt a part of a greater whole that extends far beyond me.
    b. Muje aisa anubhava hua jaise maine apni khudki pahchan kho di hai aur ek mahan sampurnata ka hissa ban chuka hun.
    c. **Intended Interpretation**: The loss of individual identity involves a blurring or disappearance of the boundaries between the self and everything else. Such experiences may be positive or negative. When experienced as positive, this experience may approximate Stace's definition of a mystical experience.
    d. **Sources**: AANEX, TAS, EDI
    e. **Iterations**: 4 in English; 6 in Hindi

f. **Issues**: The experience is rare in the general population and left people who said "no" often guessing what we meant. After four iterations, we found a way to word it in English that did not result in false positives but were unable to do so in Hindi.

2. **Unity.**
   a. I have had an experience in which it seemed as if I became one with a greater whole.
   b. Muje aisa anubhav hua hai jaise mein ek mahan sampurnata ka hissa bankar vileen ho gaya/gayi hun.
   c. **Intended Interpretation:** Unity involves the sense of becoming one with a larger whole and, thus, a blurring or disappearance of the boundaries between the self and something larger. It can be anything from a group to the cosmos. Such experiences may be positive or negative. This item is adapted from the mysticism scales. When experienced as positive, this experience may approximate Stace's definition of a mystical experience, which he defines as "an experience of undifferentiated unity," but that is not a requirement. Because this item leaves out any reference to changes in self or identity, it is vague and we can expect a wide range of responses.
   d. **Sources**: MS, MEQ, DSES
   e. **Iterations**: 4 in English, 7 in Hindi.
   f. **Issues**: The original item was interpreted broadly as including social phenomena, such as conscious conformity and the effects of crowd psychology, particularly in India. We attempted to revise the item to focus on changes in sense-of-self. We tried substituting nonordinary power/presence for "a greater whole" and also tried combining elements of Unity and Connectedness (All) to capture the boundary dissolution and mystical-type experiences. None of our efforts produced consistent interpretations in both contexts.
3. **All Disappears.**
   a. I have had an experience in which everything seemed to disappear from my mind until nothing remained.
   b. Mujhe aise laga mere dimag se sab kuch gayab ho chuka hau aur sirf khalipan bach gaya hai.
   c. **Intended Interpretation**: An experience in which the mind is empty of all content, including memories, thoughts, emotions, and sensations. Based on what we have seen initially, an actual (yes response) should describe the disappearance of the autobiographical self, i.e., any sense of who one is, where one is, absence of autobiographical memory (muscle memory is OK). Emotions are OK. If people are giving hypothetical examples, we can give the benefit of the doubt if the context seems plausible, e.g., meditation, drugs.
   d. **Sources**: MS
   e. **Iterations**: 3 in English, 2 in Hindi.
   f. **Issues**: The experience is rare in the general population and left those who said "no" guessing at what we meant.
4. **NonExistence.**
   a. I have felt as if I no longer existed.
   b. Muje anubhav hua hai jaise mera apna astitva hi nahi raha.
   c. **Intended Interpretation:** According to Lindahl & Britton, changes at this basic level of self can be very difficult to describe, and can include references to "not being there," "disappearing," or "not existing." Parnas et al. [23] define "Diminished Sense of Basic Self" as follows: "A pervasive sense of inner void, lack of inner nucleus, a pervasive lack of identity, feelings of being anonymous, as if non-existent or profoundly different from other people (this difference may sometimes be specified as difference in the worldview, being linked to an existential orientation that is fundamentally different than that of fellow humans).
   d. **Source**: EDI
   e. **Iterations**: 2 in English, 1 in Hindi
   f. **Issues**: As a change in the basic level of self, this is a rare occurrence; respondents tended to interpret it in terms of social isolation and/or not being noticed.
5. **Animated Life.**
   a. I have experienced the physical world as seemingly conscious or animated with life.
   b. Maine aisa vishishta anubhav kiya hai ki yah poora bhautik jagat jivant aur sachet hai.

c. **Intended Interpretation**: This item is derived from the concept of animism, which is the perception that all things—animals, plants, rocks, rivers, weather systems, human handiwork and perhaps even words—are animated and alive. "The perception that all things are animated and alive" is ambiguous and could refer either to a belief or an experience. We are asking whether people have experienced the physical world as animated or alive. The mysticism scales ask people if they have "felt as if all things were alive" or "seemed to be aware" or "seemed to be conscious" or contained "a living presence."
d. **Source**: MS, MEQ
e. **Iterations**: 2 each in English and Hindi.
f. **Issues:** We interpreted this item, which was derived from the mysticism scales, as referring to Animism. Some respondents interpreted it much as they did the Unity item. We replaced this item with items that more accurately capture Animism, i.e., Places [animated] and Objects [animated], and continued trying to validate the Unity item.

Other relatively rare items.

The item wording is given for the first iteration in the validation process. For the wording of subsequent iterations, please contact the authors.

6. **Synesthesia (formerly Sound Changes Things)**.
   a. I have had an experience in which different senses blended together, e.g., sounds had colors.
   b. Mujhe aisa anubhav hua hai jaise indriya ek dusre se mix ho gayi hai (jaise rango se awaj sunai de rahi hai.)
   c. **Intended Interpretation:** Synesthesia is "a phenomenon in which a person experiences a sensory stimulus, such as hearing a piece of music, and another of their senses simultaneously perceives the stimulus" (https://www.betterhelp.com/advice/synesthesia/the-many-types-of-synesthesia-explained)**.**
   d. **Iterations:** 2 in English, 2 in Hindi
   e. **Issues:** This is a rare experience for which we were not able to find a good generic description, especially for India where people may be less familiar with the concept.
7. **Hyper-Real.**
   a. I have experienced the world as different or altered, such that it somehow seemed much more "real" or vivid.
   b. Mujhe aisa anubhav hua jaise ajoobajoo sab kuch adhik vaastavik aur tivra mahsoos hone laga tha.
   c. **Intended Interpretation**: The person reports an experience in which everything is perceived as unusually vivid. The person can be awake or dreaming. We are not looking for "vivid" memories or deep insights, but for perceptual shifts in which everything seems more vivid, perhaps analogous to everything appearing in "high definition" as opposed to "normal resolution." There is potential here for responses that involve metaphoric changes in perception, e.g., "after he said that, I saw everything in a new light," which is NOT what we want. Respondents do not have to explicitly say their experience involved a change in literal (rather than metaphoric) perception, but we need to be able to infer it from the context or description.
   d. **Source**: AANEX
   e. **Iterations**: 5 in English, 4 in Hindi
   f. **Issues**: We got a spectrum of responses (e.g., clearing mind, flashbulb memory) with the initial wording; after rewording it to focus on sensory vividness, we still got mixed responses.
8. **Smell**.
   a. I have smelled a scent, fragrance, or odor even though nothing around me could have produced it.
   b. Maine ek khushboo ya gandh ka anubhav kiya hai jiska koi sadharan karan nahi tha.
   c. **Intended Interpretation:** The individual recalls an occasion in which they smelled something for which there seemed to be no ordinary cause (i.e., no external stimulus). It should have the qualities of a regular sensory perception regardless of whether they believe it was real or not. The subject may smell it alone or with others.

d. **Source**: PAGE-R, SAE
e. **Issues**: This is the least common form of hallucination; respondents confused it with memories of previous smells and new smells of an unknown source.

Items we were unable to ground in specific bodily sensations in both contexts.

The item wording is given for the first iteration in the validation process. For the wording of subsequent iterations, please contact the authors.

9. **Telepathy**.
    a. I have had an experience in which it seemed that I was able to communicate by thought alone.
    b. Muje aisa anubhav hua hai jaise maine keval vicharo dwara (muh se bole bina) kisi aur se baat-chit ya vartaalaap kiya hai.
    c. **Intended Interpretation.** Communication with another person by thinking rather than by using words or other signals. Sending and/or receiving both count.
    d. **Source**: AANEX
    e. **Iterations**: 2 in English, 2 in Hindi
    f. **Issues**: The responses included communication via passive nonverbal cues; we tried to reword to exclude this interpretation but were not successful.
10. **Energy.**
    a. I have experienced flows of energy within my body, for which there seemed to be no ordinary explanation
    b. Maine apne andar asadharan urja ya shakti ke pravah ka anubhav kiya hai
    c. **Intended Interpretation.** An experience of energy that feels like an electric current flooding through the body.
    d. **Source:** KS
    e. **Iterations**: 4 in English, 2 in Hindi
    f. **Issue**: We had difficulty anchoring this in a consistent bodily sensation. Responses in the US ranged from moods to adrenaline rushes to seizures. Responses in India, where energy was translated as *shakti,* reflected the range of meanings of that key term, including energy in the secular sense (with responses similar to those in the US) and various forms of "sacred energy" associated with deities and/or unusual human powers. Review of discussions of the "kundalini experience," which we had hoped to capture with this item, surfaced a wide range of associated phenomena that did not suggest a way to refine our item or the intended interpretation.
11. **Intuitions.**
    a. I have had a thought, insight, or intuition that did not seem to be my own.
    b. Mere paas aise vichaar, antardrshti, ya antargyaan hain jo aisa prateet hota tha jaise ki ve mujhme utpann nahi hue.
    c. **Intended Interpretation**: The experience has to refer to something thought-like that does not seem to be self-produced. The thoughts can seem like they are spoken aloud, i.e., experienced as a "voice in the head." The thoughts do not have to be attributed to another agent; we leave that to the appraisals. Marginal Interpretation: Something that lacked a sense of ownership as above, but was not a thought/insight/intuition (e.g., a feeling that felt like it was "not mine"). Differential Diagnosis: In 'Another Self in Body,' the other has to be explicit but not in this case.
    d. **Notes**: This item is derived from "thought insertion," which is defined by the ICD-10 as "feeling as if one's thoughts are not one's own, but rather belong to someone else and have been inserted into one's mind." Ratcliffe and Wilkinson [24] contend that "thought insertion involves experiencing thought contents as alien." As they elaborate: "One's experience of p is perception-like, in that p appears non-self-produced. But it also remains thought-like, in that p continues to resemble thought content more so than sensory perceptual content."
    e. **Source:** O-LIFE, AANEX, PAGE-R
    f. **Iterations:** 1 in English (validated); 5 in Hindi

g. **Issues**: We had difficulty anchoring this in a consistent sense of "non-ownership," especially in India. Many responses in India referred to a "power" that few possess, which seems to be tied to the "intuition" word in Hindi. Eliminating the Hindi word for intuition and inserting "definitely" before "feels" did not solve the problem.

12. **Moods.**
    a. I have had a mood, feeling, or emotional state that did not seem to be my own.
    b. Maine aisi bhavnae mahsoos ki hai to mere mei nahi lekin vastav mei kahin aur se utpann hui thi.
    c. **Intended Interpretation:** The experience has to refer to something emotion-like that does not seem to be self-produced. The emotions can be acted out, as in a person "overtaken by rage." The emotions do not have to be attributed to an other; we leave that to the appraisals. Marginal interpretation: Something that does not seem to be self-produced but is not a mood, feeling, or emotion (e.g., a thought).
    d. **Source:** AANEX, PAGE-R
    e. **Iterations:** 4 in English; 1 in Hindi
    f. **Issues**: We had difficulty anchoring this in a consistent sense of "non-ownership," especially in the U.S.

13. **Inner Dialogues.**
    a. I have had an inner dialogue or conversation between different aspects of myself.
    b. Muje anubhav hau hai jaise mein apne hi bhintar ek alag vyaktitva se baat-chit kar raha tha.
    c. **Intended Interpretation**: I have had inner dialogues or conversations between different aspects of myself. Internal conversations in which the aspects of the self assume different 'voices' or 'persona.' The aspects of the self must speak in their own "voice" and must engage in a conversation or dialogue. The aspects of self can be positive or negative, sought or unsought, welcome or unwelcome. The "aspect of self" is internal, but respondents may also believe the "aspects" to extend beyond the self, but that determination is left to the appraisals. Related Items: There is potential overlap between this item and "Another Self in Body," particularly with the sense of being internally divided, expressed in terms of a "divided mind" or "being of two minds." In this case, however, the divisions in the sense of self must have their own voice and engage in conversation. In so far as the aspect of the self is experienced as an other agent in their body, there can be overlap with Guidance (if the conversation involves guidance), with Messages (if the other delivers a message in the context of the conversation).
    d. **Source**: This item was inspired by Jungian inner dialogues.
    e. **Iterations**: 2 in English, 3 in Hindi
    f. **Issues**: We hoped to anchor this item in voices that seemed to have their own sense of agency or autonomy, but were unable to arrive at wording that separated those sorts of inner voices from competing thoughts and pro/con lists.

14. **Altered Time.**
    a. I have had an experience in which time seemed to slow down, or speed up, or cease to exist.
    b. Mujhe aisa anubhav hua jaise samay dhima ho gaya hai athva samay sthir ho gaya hai.
    c. **Intended Interpretation:** Experiences are somewhere along the spectrum of being shocked and no longer noticing time, and the time dilation felt in a life-threatening car crash.
    d. **Source**: AEI, AANEX, NDES, MEQ
    e. **Iterations**: 3 in English, 8 in Hindi
    f. **Issues**: The item as initially worded overlapped too much with absorption. We were able to validate a modified, more focused version in English, but 3/6 'yes' responses were not understood in India so the item failed to validate in both contexts.

Evidence of Validity Questioned.

We collected evidence of validity for this group of items, but upon final review, we identified issues that made the evidence less compelling and led us to leave the item out of the validated INOE. The item wording is given for the final iteration in the validation process.

15. **Auras.**
    a. I have seen an aura or halo of light surrounding a person, for which there seemed to be no ordinary source.
    b. Maine ek vyakti ke ird-gird tej ya prakash dekha hai jiski koi sadharan vajah nahi thi.
    c. **Intended Interpretation**: The individual recalls an occasion in which they saw a light or an energy field around /surrounding a person for which there seemed to be no ordinary cause (i.e., no external stimulus). The light or energy field may include the person's body or a portion thereof, such as their face, but it has to have the qualities of a visual perception regardless of whether they believe it was real or not. The subject may see it alone or with others. Gurus and saints have them (allegedly).
    d. **Source:** AEI, AANEX, KS, SAE
    e. **Iteration Count:** 2 in English, 2 in Hindi
    f. **First Iteration**: I have seen an aura or light glowing around a person, for which there seemed to be no ordinary source. / Maine ek vyakti ke ird-gird ek chamak ya prakash dekha hai jiski koi sadharan vajah nahi thi.
    g. **Reason for Elimination:** There was only one 'yes' response in the US and it was not understood.
16. **Other Reality.**
    a. I have been in contact with what seemed to be a world or reality other than this world.
    b. Mai is duniya (jahan saare log samanyaroop se rahte hai) se ekdum hi alag duniya ke sampark mei hun.
    c. **Intended Interpretation:** This can include what seems like travel to another world, e.g., another planet, heaven, an astral plane, the spirit world, or contact with an entity that presumably came from another world, such as an alien, a ghost, spirit, or dead person. The "world" should not be part of "this world," as it is in "we are worlds apart in our thinking" or "we live in separate worlds." As long as it is not part of "this world," the other world can be appraised as real or imaginary -- we leave that to the appraisals.
    d. **Source:** AEI, NDES
    e. **Iterations:** 2 in English, 2 in Hindi
    f. **First Iteration**: I have been in contact with what seemed to be a world or reality other than the one I usually inhabit. / Mai jis duniya mei samanya rupse rahta hun us duniya se ekdam hi alag duniye ke sampark mei hun.
    g. **Reason for Elimination:** The intended interpretation and responses were quite varied and in retrospect we decided that this item was not well anchored in bodily sensations, feelings, or perceptions.
17. **Objects Moving.**
    a. I have noticed objects that seemed to move by themselves, disappear or appear, or change their appearance (not caused by technology).
    b. Maine aisi asamanya vastue dekhi hai jo kisi bhi technology dwara sanchalit nahi hai lekin apne aap hi chalu-bandh hoti hai, ya khudse hi chalti-rukti hai, ya dikhai-gayab hoti hai, ya fir achanak aakar-swarup badalti hai.
    c. **Intended Interpretation:** This item is intended to capture movement or changes in objects that have no obvious cause. Such claims are sometimes asserted as evidence of miracles, spirits/ghosts, the paranormal, etc. Siri or Alexa turning things on and off does NOT count!
    d. **Source:** AEI, KS, PAGE-R, SAE
    e. **Iterations:** 2 in English, 2 in Hindi
    f. **First Iteration**: I have noticed items, objects, or devices that seemed to move by themselves, turn on or off, disappear or appear, or change their appearance or their form. / Maine aisi asamanya vastue ya upkaran dekhe hai jo apne aap hi chalu-bandh hoti hai, ya khudse hi chalti-rukti hai, ya dikhai-gayab hoti hai, ya fir achanak aakar-swarup badalti hai.

g. **Reason for elimination**: The interpretations looked good, but our 3 Yes-responses were not understood (one in Hindi was marked as understood, but they seem to be recounting a legend). We get some of what we wanted to get with this item with Objects (animated).

18. **Meaninglessness**.
    a. I have had an experience in which it felt as if my life had no meaning or purpose.
    b. Muje aisa anubhav hua ki mere jivan ka koi matlab nahi hai.
    c. Intended Interpretation: An experience in which life feels pointless or meaningless, unfulfilling, without direction, goals, purpose, usefulness, or companionship. May be associated with feeling hopeless or like a failure. Note: Nihilists, material is, and humanists may also say yes to this item because they believe life is inherently meaningless, even though they do not report any specific feelings as described above; 2/20 (2/8 yes responses) were of this type on MTurk.
    d. **Source:** AANEX
    e. **Iterations:** 2 in English, 2 in Hindi
    f. **First Iteration**: I have had an experience of meaninglessness that stood out from all other such experiences. / Mujhe nirtarthakta/arthahinata/vyarthata ka anubhav hua he jo baki aise anubhavo se alag tha.
    g. **Reason for Elimination:** This item doesn't seem to be as well anchored as Hopelessness. The responses tend to overlap except for those who ignored the "felt experience" part and said 'yes' because they believe there is inherently no meaning. Examples weren't as specific here as with hopelessness. If we had refined it (as we later did some other items) to read, e.g.: "I have had a specific experience in which it felt as if …", we might well have been able to validate it.
19. **Another Self in Body.**
    a. I have had an experience in which it seemed like there was another self or being in my body.
    b. Muje aisa anubhav hua jaise mere andar mere sivaay koi anya atma ya jeev hai.
    c. **Intended Interpretation**: This item includes any experience in which it seems as if an "other" (not "me") is present in the body. The other can be associated with self-alien movements, thoughts, or emotions. It may be minimally or robustly realized. The other may be experienced as co-conscious, i.e., both present at the same time, or as alternating. This item is worded to capture a range of agentic divisions from a sense of divided mind to dissociative identity disorder (aka multiple personalities/alter personalities) and complete or partial 'possession' by spirits or deities. The reality of the other agent, whether it's a split off part of themselves or an external agent, is all part of the appraisal.
    d. **Source:** AEI, PAGE-R
    e. **Iterations:** 3 in English, 2 in Hindi
    f. **First Iteration**: I have had an experience in which it seemed like there was another self or being in me. / Muje aisa anubhav hua jaise mere andar koi anya atma ya jeev hai.
    g. **Reason for Elimination.** During the validation of this item, it was rarely endorsed in either population. Participants who said 'No' gave a range of hypothetical examples that matched the intended interpretation. Most of the 'Yes' responses described a co-conscious, conscience-like 'inner voice' and/or a sense of inner conflict or being 'of two minds.' Although the intended interpretation allowed for a range of self-alien movements, thoughts, or emotions, we decided during the final review that this item posed difficulties much like those we encountered with Inner Dialogues, Moods, and Intuitions (discussed above). Here too we had hoped to anchor this item in selves or beings that seemed to have their own sense of agency or autonomy, but we were unable to arrive at wording that separated those sorts of inner voices from more mundane inner conflicts. For other issues related to this item, see (25).
20. **Connectedness (others).**
    a. I have had an experience in which I became one with everyone at a large group event and lost my sense of individuality.
    b. Jab kisi ek sammelan/utsav mei logo ke bade samooh ke saath muje aisa anubhav hua hai jaise mein us samooh ke saath ek-ras ho gaya/gayi hun aur meri khudki pahchan meet gayi hai.

c. **Intended Interpretation**: An experience of losing oneself (to some degree) in a group or collectivity. It typically involves the blurring or even dissolution of self-other boundaries.

d. **Notes**: This corresponds to Durkheim's notion of "collective effervescence." Examples would include all kinds of group events, such as worship, group singing, sports events, concerts, rallies, crowds, etc. The experience can be positive or negative. Based on Yaden et al's [26] definition of self-transcendent experiences as "transient mental states marked by decreased self-salience and increased feelings of connectedness" with others or one's surroundings, this falls at the more intense end of the spectrum. In the U.S., when people appraised the experience negatively, they tended not to understand it as intended. They describe consciously conforming, which they resent; they still believe they are an individual but think that others don't recognize their individuality. 80% understood - weaker item.

e. **Source**: This item was inspired by items that referred to a feeling of connection to all life in the DSES and a sense of union with others in the EDI and refined in light of Durkheim's notion of collective effervescence.

f. **Iterations:** 4 in English, 4 in Hindi

g. **First Iteration**: I felt a sense of being connected to everyone around me. / Maine aisa mahsus kiya hai jaise mei apne aas-paas ke sabhi logo ke saath juda hua hun.

h. **Reason for Elimination**. Connectedness (others) required major changes. Its wording, which initially referred to feeling a "sense of connectedness with everything around me," was revised to focus more specifically on "becoming one with everyone in the context of a group event." The final wording was understood by 95% of respondents in India, but only 80% in the US. About half of the respondents in the US said 'yes' (9/20), but of those 3 were coded as Not Understood. In two cases, we coded them as not understood because the respondent felt like individuals but weren't recognized as such in the context of a group event. This item, although better understood than the sense of self items we did not include, suffered from similar difficulties; in this case, differentiating relatively mundane experiences of loss of self due to conscious conformity and/or lack of recognition by others from the loss of sense of self that can occur in crowds, sports events, or intense rituals.

21. **Goosebumps (formerly Bodily Sensations).**

a. I can recall a <u>specific</u>, intense experience in which all of my body hairs suddenly stood on end (not due to cold or illness).

b. Mujhe ek <u>vishishta aur alag</u> hi anubhav hua hai jahan mere sharir ke saare rongte khade ho gaye ho. (<u>thand ya bimari ke karan nahi</u>).

c. **Intended Interpretation**: The person has an experience in which "small bumps appear on the surface [of their skin] as the hairs become erect" (technically known as piloerection, frission, cutis anserina, and horripilation) (adapted from Oxford Language online).

d. **Notes**: They need to recall a specific experience. We rule out cold (the most common cause) and illness and are looking for other sorts of experiences e.g., shocking, frightening, awe-inspiring, or extremely exciting.

e. **Source**: This item was inspired by items that referred to feeling unusual sensations in the AANEX and KS.

f. **Iterations:** 4 in English, 4 in Hindi.

g. **First Iteration**: I have had an experience of goosebumps or my hairs standing on end (when I was not cold) that stood out from other such experiences. / Mujhe ek alag hi anubhav hua hai jahan mere sharir ke saare rongte khade ho gaye ho.

h. **Reason for Elimination**. The final wording was understood by 95% of respondents in India, but only 80% in the US. The PPU for the US was 69%, because 4/13 'yes' respondents did not have a specific experience or type of experience in mind.

22. **Presence (dead).**

a. I have sensed the presence of, or interacted with, a person who was dead, either directly or with the assistance of another person.

b. Khudse ya anya vyakti ki madad se, maine kisi mrit vyakti se sampark ya upasthiti ka anubhav kiya hai.

c. <u>Intended Interpretation</u>: The sense that a person who was once alive is now present based on some sort of perceived internal or external cues.

d. Notes: The subject may or may not interact with the perceived presence. The presence may be characterized as a known person, e.g., a dead friend or relative, or more vaguely as a ghost, spirit, ancestor. Any characterization is OK as long as they were once alive in human form. The perceived presence can be positive or negative, sought or unsought, welcome or unwelcome. The deceased may manifest through a living being or material object (e.g., an 'oracle,' 'medium,' 'shaman', a 'spirit guide', or 'talisman'). The power or being may simply seem to be present; whether the subject actually thinks it is present will come out in the appraisals.
e. Differential diagnosis: Signs or messages without the sense of an "other" being present fall under our **Messages** item.
f. Related Items: There is potential overlap between this item and other Presence-related items in so far as the "other" is viewed as having previously been alive (embodied).
g. Source: This item was inspired by items that referred to communicating with the dead in the AEI, PAGE-R, SAE, and NDES.
h. **Iterations:** 2 in English, 2 in Hindi.
i. **First Iteration**: I have sensed the presence of, or interacted with, a person who was missing or dead, either directly or with the assistance of another person. / Khudse ya anya vyakti ki madad se, maine kisi mrit ya lapata vyakti ka sampark ya upasthiti ka anubhav kiya hai.
j. **Reason for Elimination**. The final wording was understood by 95% of respondents in the US and 86% of respondents in India; the PPU/NPU for the US were 100%/95%, the PPU/NPU for India were 50%/94%. The 50% PPU in India was due to 2/4 respondents who said 'yes' describing a deity or spirit rather than a dead person. The 100% PPU in the US was based on only one response. Despite the high overall proportion of respondents who understood the item as intended, this item was removed due to the risk of skewed cross-cultural comparisons, where the prevalence in India is likely to be inflated relative to the prevalence in the US. This item requires further testing in the US and a rewrite in India to make sure people focus on the dead rather than deities/spirits.

Validation Not Attempted

We developed 26 additional items, 23 of which were used in an exploratory study [27], and 3, which were developed later. We did not attempt to validate them either because we viewed them as low priority for a comparative study of the US and India, discovered wording problems in the English items, had problems translating them into Hindi, and/or decided that an item was redundant.

Lower Priority

Most of the items we designated as low priority were rare and/or rarely valorized in the US and/or India.

1. **Dread.** I have had an experience of dread that stood out from all other such experiences.
2. **Humor.** I have had an experience of humor or a fit of laughter that stood out from all other such experiences.
3. **Taste.** I have perceived a taste for which there seemed to be no obvious cause.
4. **Flashbacks.** I have experienced images from my distant memory that stood out from all other such experiences.
5. **Body Changes**. I have detected changes in my body for which there seemed to be no obvious cause.
6. **Bodily Distortions.** I have experienced changes in the size or shape of my body for which there seemed to be no obvious cause.
7. **Draft.** I have felt a draft or a sudden change in temperature, for which there seemed to be no obvious cause.

8. **Immunity.** I have had the experience of being seemingly immune or invulnerable to harmful objects or environments (e.g., toxins, poisons, or fire).
9. **Memory Gaps**. I have experienced gaps in my memory such that I couldn't remember what I had been doing moments ago.
10. **Mental Imagery.** I have had mental images while awake that seemed as if they were really happening in the external world.
11. **Presence (visual).** I have seen patterns, figures, colors, or shapes for which there seemed to be no ordinary cause.
12. **Monitored.** I have had an experience in which I felt monitored or watched, or otherwise the subject of external attention, when there was no obvious evidence for this.
13. **Psychic Vampirism.** I have had the feeling of gaining or losing energy in my body when interacting with certain people, objects, or entities.
14. **Faces (distorted).** I have seen the face of someone else or the reflection of my own face change or distort.
15. **Teleportation.** I have had an experience in which it seemed as if I was suddenly in a different physical location without having moved at all.
16. **Mental Control.** I have had an experience in which it seemed that I caused changes in people or objects through the power of thought.
17. **No Body.** I have felt as if I no longer had a physical body.
18. **Hypo-Real**. I have experienced the world as different or altered, such that it somehow seemed much less "real" (e.g., flat, artificial, or illusory).

Translation or Wording Problems

19. **Warmth.** I have felt a sudden sense of warmth within myself for which there seemed to be no obvious cause. [Translation problems in Hindi. Warmth has very different connotations in India than the U.S.]
20. **Space**: I have had an experience in which space seemed to change or distort. New. [Translation problems in Hindi.]
21. **Immanent Karma (+/-).** When something especially good has happened, I felt as if something bad was bound to happen (or the other way around). [This item is an appraisal of the "good" or "bad" experience rather than an experience in its own right.]
22. **Ineffability.** I have had an experience that was very difficult if not impossible to put into words. [This item is appraisal related, i.e., not tied to any specific experience; it is typically an experience one has after the experience that one is trying to put into words.]
23. **Surrender:** I have had the experience of letting go, ceasing all effort, and turning things over to something greater than myself. New. [This is a precursor to experience but not an experience in its own right.]
24. **Paradox**. I have had an experience of two opposing feelings or thoughts or actions suddenly resolved into something new or different. New. [This item is difficult to narrow down and make specific.]

Redundant

25. **OBE2.** I have had an experience in which it seemed as if I was looking at my physical body from outside. [This item is too specific; OBE is enough.]
26. **Interaction.** I have interacted with what seemed to be non-ordinary forces or beings. [Deleted due to overlap with other presence items.]

References

1. Gallagher C, Kumar VK, Pekala RJ. The Anomalous Experiences Inventory: Reliability and validity. J Parapsychol. 1994;58:402–28.

2. Brett CMC, Peters EP, Johns LC, Tabraham P, Valmaggia LR, McGuire P. Appraisals of Anomalous Experiences Interview (AANEX): A multidimensional measure of psychological responses to anomalies associated with psychosis. Br J Psychiatry Suppl. 2007;51:s23-30.

3. Drinkwater K, Denovan A, Dagnall N, Parker A. The Australian Sheep-Goat Scale: An evaluation of factor structure and convergent validity. Front Psychol. 2018;9:1594.

4. Thalbourne MA, Delin PS. A new instrument for measuring the sheep-goat variable: Its psychometric properties and factor structure. J Soc Psych Res. 1993;59(832):172–86.

5. Merckelbach H, Horselenberg R, Muris P. The Creative Experiences Questionnaire (CEQ): A brief self-report measure of fantasy proneness. Personal Individ Differ. 2001 Oct;31(6):987–95.

6. Underwood LG, Teresi JA. The Daily Spiritual Experience Scale: Development, theoretical description, reliability, exploratory factor analysis, and preliminary construct validity using health-related data. Ann Behav Med. 2002;24:22–33.

7. Bernstein E, Putnam FW. Development, reliability, and validity of a dissociation scale. J Nerv Ment Dis. 1986;174:727–35.

8. Nour MM, Evans L, Nutt D, Carhart-Harris RL. Ego-dissolution and psychedelics: Validation of the Ego-Dissolution Inventory (EDI). Front Hum Neurosci. 2016;10:269.

9. Thalbourne MA. The Kundalini Scale and its relation to mystical and paranormal experience. In: Fallio VW, editor. New Developments in Consciousness Research. Nova Science Publishers; 2007. p. 119–31.

10. Barrett FS, Johnson MW, Griffiths RR. Validation of the revised Mystical Experience Questionnaire in experimental sessions with psilocybin. J Psychopharmacol. 2015 Nov;29:1182–90.

11. MacLean KA, Leoutsakos JMS. MS, Johnson MW, Griffiths RR. Factor analysis of the mystical experience questionnaire: A study of experiences occasioned by the hallucinogen psilocybin. J Sci Study Relig. 2012;51(4):721–37.

12. Hood RW. The construction and preliminary validation of a measure of reported mystical experience. J Sci Study Relig. 1975;14:29–41.

13. Greyson B. The Near-Death Experiences Scale: Construction, reliability, and validity. J Nerv Ment Dis. 1983;171(6):369–75.

14. Granqvist P, Hagekull B. Seeking security in the New Age: On attachment and emotional compensation. J Sci Study Relig. 2001 Sep;40(3):527–45.

15. Fach W, Atmanspacher H, Landolt K, Wyss T, Rössler W. A comparative study of exceptional experiences of clients seeking advice and of subjects in an ordinary population. Front Psychol. 2013;4:65.

16. Tobacyk JJ. A Revised Paranormal Belief Scale. Int J Transpers Stud. 2004 Jan 1;23(1):94–8.

17. Irwin HJ, Dagnall N, Drinkwater K. Parapsychological experience as anomalous experience plus paranormal attribution: A questionnaire based on a new approach to measurement. J Parapsychol. 2013;77(1):39–53.

18. Lange R, Ross RM, Dagnall N, Irwin HJ, Houran J, Drinkwater K. Anomalous experiences and paranormal attributions: Psychometric challenges in studying their measurement and relationship. Psychol Conscious Theory Res Pract. 2019 Dec;6(4):346–58.

19. Jamieson GA. The modified Tellegen Absorption Scale: A clearer window on the structure and meaning of absorption. Aust J Clin Exp Hypn. 2005;25.

20. Tellegen A, Atkinson G. Openness to absorbing and self-altering experiences ("absorption"), a trait related to hypnotic susceptibility. J Abnorm Psychol. 1974;83(3):268–77.

21. Lange R, Thalbourne MA, Houran J, Storm L. The Revised Transliminality Scale: Reliability and validity data from a Rasch top-down purification procedure. Conscious Cogn. 2000;9(4):591–617.

22. Mason O, Claridge G. The Oxford-Liverpool Inventory of Feelings and Experiences (O-LIFE): Further description and extended norms. Schizophr Res. 2006;82(2–3):203–11.

23. Parnas J, Møller P, Kircher T, Thalbitzer J, Jansson L, Handest P, et al. EASE: Examination of Anomalous Self-Experience. Psychopathology. 2005;38(5):236–58.

24. Ratcliffe M, Wilkinson S. Thought insertion clarified. 2017;246–69.

25. Taves A, Barlev M. A feature-based approach to the comparative study of "nonordinary" experiences. Am Psychol. 2023 Jan;78(1):50–61.

26. Yaden DB, Haidt J, Hood RW, Vago DR, Newberg AB. The varieties of self-transcendent experience. Rev Gen Psychol. 2017;21(2):143–60.

27. Barlev M, Taves A, Kinsella M. Mapping nonordinary experiences across cultures in the U.S. and India. [Preprint]. 2021. Available from https://doi.org/10.31234/osf.io/fvc6w
